# Reducing DNA context dependence in bacterial promoters

**DOI:** 10.1371/journal.pone.0176013

**Published:** 2017-04-19

**Authors:** Swati B. Carr, Jacob Beal, Douglas M. Densmore

**Affiliations:** 1 Molecular Biology, Cell Biology & Biochemistry, Boston University, Boston, MA, United States of America; 2 Raytheon BBN Technologies, Cambridge, MA, United States of America; 3 Electrical & Computer Engineering, Boston University, Boston, MA, United States of America; Imperial College London, UNITED KINGDOM

## Abstract

Variation in the DNA sequence upstream of bacterial promoters is known to affect the expression levels of the products they regulate, sometimes dramatically. While neutral synthetic insulator sequences have been found to buffer promoters from upstream DNA context, there are no established methods for designing effective insulator sequences with predictable effects on expression levels. We address this problem with Degenerate Insulation Screening (DIS), a novel method based on a randomized 36-nucleotide insulator library and a simple, high-throughput, flow-cytometry-based screen that randomly samples from a library of 4^36^ potential insulated promoters. The results of this screen can then be compared against a reference uninsulated device to select a set of insulated promoters providing a precise level of expression. We verify this method by insulating the constitutive, inducible, and repressible promotors of a four transcriptional-unit inverter (NOT-gate) circuit, finding both that order dependence is largely eliminated by insulation and that circuit performance is also significantly improved, with a 5.8-fold mean improvement in on/off ratio.

## Introduction

The composability of complex genetic devices from well-characterized, basic DNA parts is a central premise of synthetic biology [1]. This principle has been a driving force behind the creation of modular genetic devices and their curation into community repositories (e.g., [2–4]), as well as the development of computational tools for predictive design (e.g., [5–8]). However, *a priori* genetic regulatory network design continues to be a major challenge [1, 9]: at present, most rationally designed genetic regulatory networks fail to perform well when tested [10]. Instead, successful genetic regulatory networks are often arrived at by repeated iterations or by combinatorial construction and screening of a library of variants of the intended network, from which one or a few high-performing variants are selected [1, 11].

The poor performance of an engineered genetic regulatory network can be due to many different factors, including interactions between the various devices making up the network, poorly understood interactions of devices with their operating environment, and physical or functional interactions between engineered components and the host genetic machinery [10, 12]. In this work, we focus specifically on the effect of local genetic context on device performance, recently identified as a leading cause of genetic regulatory network design failures [12], and in particular on the sensitivity of bacterial promoters to the DNA sequence upstream of their 5’ end. This is a critical issue because in any genetic construct containing more than one transcriptional unit, the DNA sequence upstream of at least some of the promoters will be affected by the choice and arrangement of parts in other transcriptional units.

Moreover, the effect of upstream DNA sequence can be quite strong, particularly for the minimal promoters often favored in synthetic biology, which are often only a few dozen base-pairs long. Binding of the *α*-subunit (*α*-CTD) of the RNA polymerase (RNAP) to A/T-rich Upstream Promoter (UP) elements placed between -40 and -60 bp of the transcription start site (TSS) can increase transcription by 30-60x [13], the RNAP *α*-CTD has been reported to bind to DNA sequences at least as far as 100 bp upstream of the TSS resulting in altered protein expression levels [14, 15], and as few as 4 bp of DNA sequence immediately upstream of minimal constitutive promoters have been observed as being sufficient to alter expression by up to 13-fold [16].

One approach to reducing the impact of upstream DNA sequence is to extend the 5’ end of the promoter with an “insulator” sequence, thereby increasing the separation of the active components of the promoter from the potentially variable portion of upstream sequence and thus hopefully reducing its impact. Davis *et al.* showed that a synthetic insulator sequence inserted at the 5’ boundary of susceptible minimal promoters did successfully inhibit increased rate of transcription by an UP element [13]. However, the insulator they tested also altered the expression levels of the promoters to an unpredicted degree, a result that we further confirm in this work: at present, there is simply no effective model available for predicting the interaction of a promoter and its upstream nucleic acid sequence. As such, any potential insulator sequence must be carefully tested in combination with the promoter being insulated, in order to ensure that the desired expression level is maintained.

To address these challenges, we have developed Degenerate Insulation Screening (DIS), a novel method for insulating promoters based on a randomized 36-nucleotide insulator library. DIS draws random samples from this library of 4^36^ potential insulated promoters in a high-throughput, flow-cytometry-based screen, comparing against a reference device for precise expression control. We test this method by applying it to insulate a minimal constitutive promoter (BBa_J23100, hereafter referred to as J23100), an inducible promoter (BBa_I13453, hereafter referred to as pBAD) and a repressible promoter (BBa_R0040, hereafter referred to as pTet). Our experiments show that using DIS-insulated promoters largely eliminates the order dependence of component transcriptional units on the performance of a four transcriptional unit genetic inverter (NOT-gate). Regularizing promoter behavior also improves circuit performance, providing an incremental increase in on/off ratio with each additional insulated promoter to an ultimate 5.8-fold mean improvement when all promoters are insulated.

## Results

Degenerate Insulation Screening (DIS) is a novel promoter insulation protocol based on amplification with degenerate primers and screening to compare against reference expression levels. This method is motivated by the fact that there are many possible mechanisms by which an upstream sequence might affect promoter expression (e.g., cryptic binding sites, secondary sequence structure), but any given such mechanism is likely to pertain to only a small fraction of sequences (e.g., those that match a cryptic binding site). We thus hypothesized that randomly generated sequences should have a reasonable probability of behaving as good insulators, but that it might also be necessary to screen many candidate sequences in order to find insulating sequences that closely match a reference expression level from an uninsulated device. Initial tests with a small number and hand-generated random DNA sequences produced results consistent with this hypothesis (S1 Fig), leading us to develop a method for rapid in-context screening of many candidate insulators to obtain a collection of insulators matching the desired reference expression level.

In the remainder of this section, we first present the DIS protocol and results showing that DIS produces libraries with a wide distribution of device behaviors, supporting selection of multiple insulator options matching to a desired reference behavior. We then validate the insulation provided by DIS promoters by application to a four transcriptional-unit inverter circuit, finding that DIS-insulated promoters largely eliminate order dependence, as well as significantly improving circuit performance.

### DIS enables matching of insulated and reference expression

Basic idea of DIS is to generate a custom set of insulators for each promoter by screening a large collection of constructs with random spacers inserted upstream of the promoter. The insulators are then those spacer constructs whose expression levels match a desired reference level of expression (As we will see, the reference level need not necessarily be taken from observations of an actual system. It is, however, more likely to succeed when this is the case, because then it is known that there is at least one upstream context that achieves the desired reference level).

**Fig 1:**
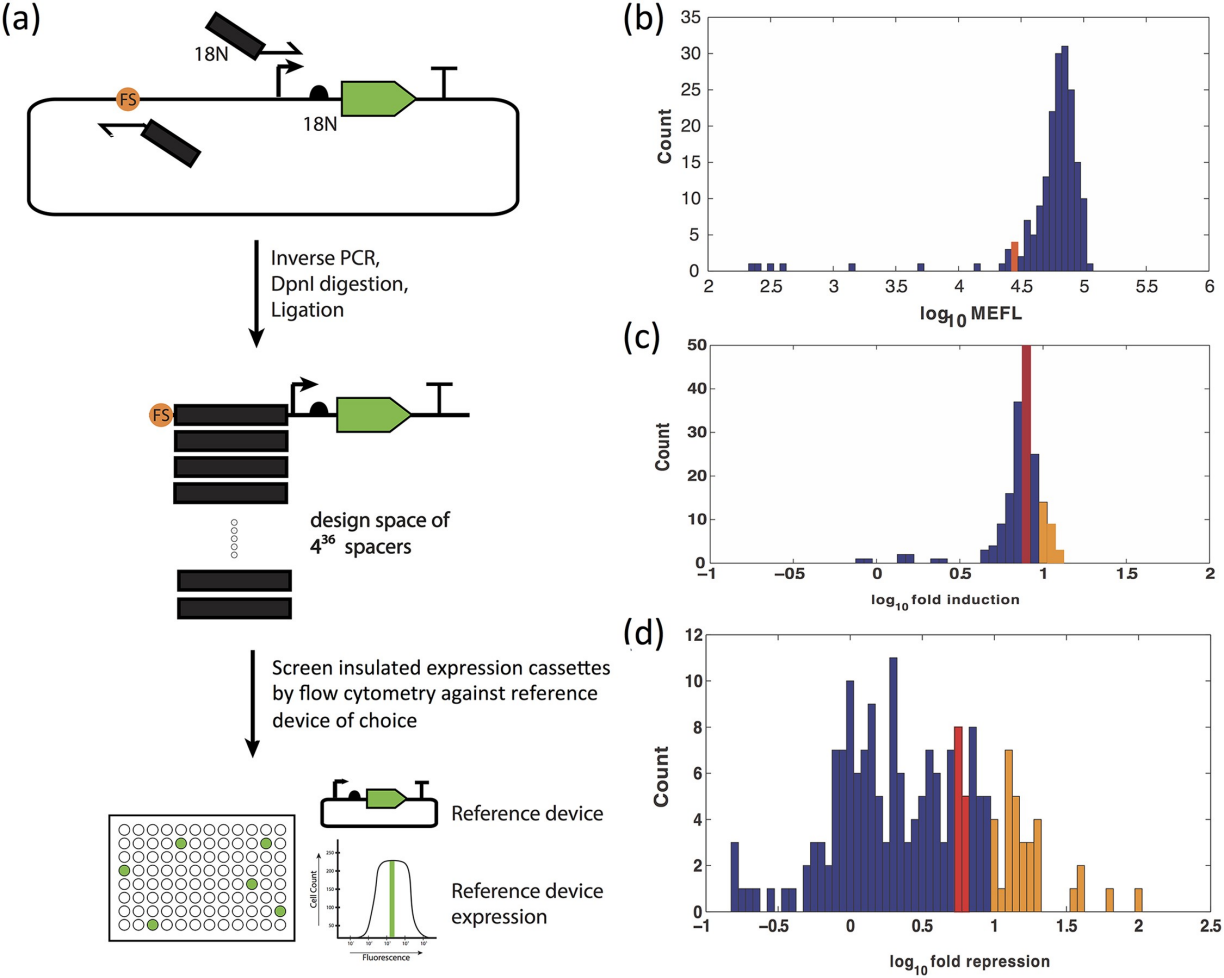
(a) Degenerate Insulation Screening (DIS) protocol for insulation of promoters. The DIS protocol produces spacers with a wide range of expression behaviors, including many matches to a given reference level and variants that can be used for tuning expression: (b) distribution of GFP expression from samples of 36 nt insulated J23100_E-GFP cassette library, in units of Molecules of Equivalent FLuorescein (MEFL); (c) distribution of fold induction of GFP expression from samples of inducible 36 nt insulated pBAD_F-GFP library upon induction with L-arabinose. (d) distribution of fold repression of RFP expression from samples of repressible 36 nt insulated pTet_A-RFP library upon induction of repressor expression with L-arabinose. Red bar indicates performance identical to reference device; orange bars mark samples with ≥10X induction/repression. Plus and minus distributions for pBAD and pTet are shown in S4 and S5 Figs, respectively.

Our implementation of this concept in the DIS protocol is shown in Fig 1(a). We begin with a simple expression cassette in which the chosen promoter controls expression of a fluorescent protein. A library of spacers upstream of the chosen promoter is generated by performing a standard, automation-friendly inverse PCR reaction using divergent forward and reverse primers ending with 100% degenerate primer extensions, followed by a blunt-end ligation to circularize the linear PCR products. The library is then transformed into *E. coli* and cultured to produce clonal populations of individual library elements, which may then be further split for testing multiple input conditions. All samples are then screened by flow cytometry to identify samples containing DNA spacers that empirically provide the precise desired expression level or levels (e.g., matching a reference sample used for characterization and modeling); these spacers are the desired library of insulators. Finally, the insulator library can be sequenced to identify effective spacer-promoter pairs and/or used directly in construction of larger systems (e.g., if the screening vectors are also appropriate for assembly). Additional details may be found in Materials and Methods and in S1 File.

To test DIS, we applied it to insulate three commonly used promoters: a constitutive minimal promoter (J23100), an inducible promoter (pBAD), and a repressible promoter (pTet). For a baseline to compare against, we tested the impact of upstream sequence on these promoters against four MoClo assembly [16, 17] fusion sites (S2 Fig), finding high sensitivity to upstream sequence in J23100 and pBAD (with means spanning a 12.5-fold and 11.8-fold range, respectively) but no significant impact on pTet (with means spanning only a 1.5-fold range). Based on prior results [13–16] and preliminary tests with several spacer lengths (S3 Fig), we selected a spacer length of 36 bp, meaning each primer used has an 18N extension. For J23100, DIS was conducted using the MoClo “E” fusion site, which had most strongly impaired expression levels in the preliminary test, and taking its strong performance with the MoClo “A” fusion site as the reference to be achieved. The pBAD promoter was most strongly impacted by the MoClo “F” fusion site, so that was used for its screening, while we selected MoClo “A” for the less-affected pTet. For both pBAD and pTet, the desired reference was set as a 10-fold change in expression level, and the promoter is evaluated in both the presence and absence of arabinose with the aid of a helper construct. For a fluorescent reporter, both J23100 and pBAD used GFP, while pTet used RFP instead. Library size was chosen to be small enough for fast execution with standard labware on two 96-well plates per condition, but large enough to obtain a good sampling of readily achievable expression levels: our execution created a library of 192 spacer-promoter constructs for each of J23100 and pTet, and 178 spacer-promoter constructs for pBAD.

The distributions of geometric mean fluorescent expression from the DIS libraries for J23100, pBAD, and pTet are shown in Fig 1(b)-1(d), respectively, with additional detail for pBAD and pTet provided in S4 and S5 Figs. As predicted, the libraries for all three promoters contain a wide distribution of device behaviors, providing many options to select between for matching a reference behavior. For the J23100 and pBAD promoters, the bulk of the distribution was confined to a single order of magnitude in range, with a small scattering of lower functionality devices, while the pTet promoter exhibited much more variability in observed behavior. We thus have evidence that DIS provides a simple means of exploring many candidate spacers in context, and may be expected to generate many options for insulators that yield expression levels closely matching a desired reference level. Finally, an obvious corollary of the range of available options in the library is the potential for tuning expression levels by selecting insulators with a different observed expression level than the original reference device.

### Promoter insulation can eliminate order dependence and improve circuit performance

**Fig 2:**
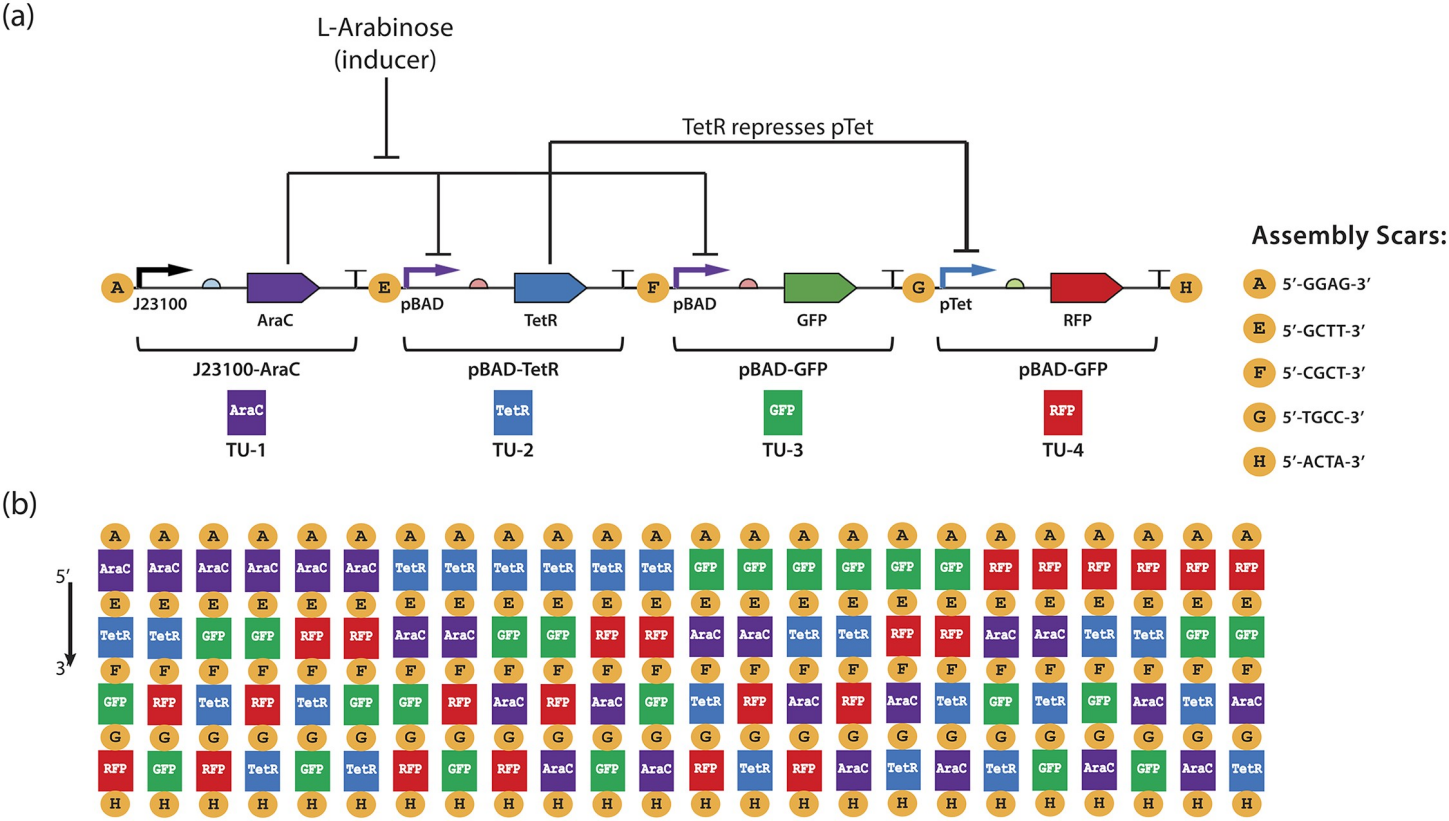
Efficacy of DIS promoter insulation is tested through permutation of a bacterial, monocistronic, transcriptional TetR-“inverter” circuit composed of 4 tandem transcriptional units assembled by means of the MoClo DNA assembly method. (a) shows the “base” design that is permuted, in which constitutive expression of AraC is induced by L-Arabinose, relieving its repression of the pBAD promoter and resulting in expression of the TetR repressor and GFP. TetR, in turn, represses the pTet promoter, reducing the expression of RFP. Thus, when “on” this circuit should have high green and low red fluorescence, and when “off” it should have low green and high red. Circles between transcriptional units represent the 4 bp scars left by MoClo assembly at the edges of each transcriptional unit in the final construct. (b) A set of 24 permutations are created, covering all possible orderings of transcriptional units, holding orientation constant. As the contents of each transcriptional unit are the same, any differences in behavior are expected to be due to differences in genetic context, and should be able to be eliminated by effective insulators.

To test the efficacy of DIS-generated insulators, we combined the three promoters to form an “inverter” circuit comprising four transcriptional units (Fig 2). In this circuit, J23100 drives strong constitutive expression of AraC. The presence of L-Arabinose induces expression from two pBAD promoters by relieving their repression by AraC: one pBAD promoter controls expression of green fluorescent protein (GFP), while the other controls expression of the TetR repressor. TetR, in turn, represses the pTet promoter, reducing the expression of red fluorescent protein (RFP). Thus, when L-arabinose is present, this circuit should express a high green and low red fluorescence, and when it is absent should express low green and high red.

We test the impact of local DNA context on circuit performance by using MoClo assembly [16, 17] to create all 24 possible orientation-preserving (i.e., same-strand) orderings of the circuit’s four transcriptional units. Since the only thing that varies in these circuits is the surrounding DNA context (MoClo fusion sites and flanking sequences), any differences in behavior may be expected to be due to those differences in context. From the DIS libraries previously generated, we selected four DIS-insulated promoters: one J23100 and two pBAD matched to their reference values (we used two different pBAD insulators to avoid encouraging recombination), and one pTet with 10-fold repression. In all cases, multiple insulated promoters matching these criteria were available, but since all matching insulated promoters were expected to perform equivalently, we made an arbitrary choice on which to use from the matching set. We constructed several variations of this permutation set using varying degrees of insulation: uninsulated, insulating all promoters with random spacers, DIS-insulating only J23100, DIS-insulating only pBAD, DIS-insulating both J23100 and pBAD, and DIS-insulating all promoters.

Each permutation set was then cultured in triplicate overnight in both the uninduced (“off”, 0 mM L-Ara) state and the induced (“on”, 1 mM L-Ara) states, and the expression levels of GFP and RFP measured by flow cytometry. Finally, we compare expression levels by calculating the geometric mean expression of each sample and taking the ratio of the “on” state over the “off” state (i.e., computing fold-change in expression). Ideally, all permutations should show similar strong levels of GFP induction and RFP repression.

**Fig 3:**
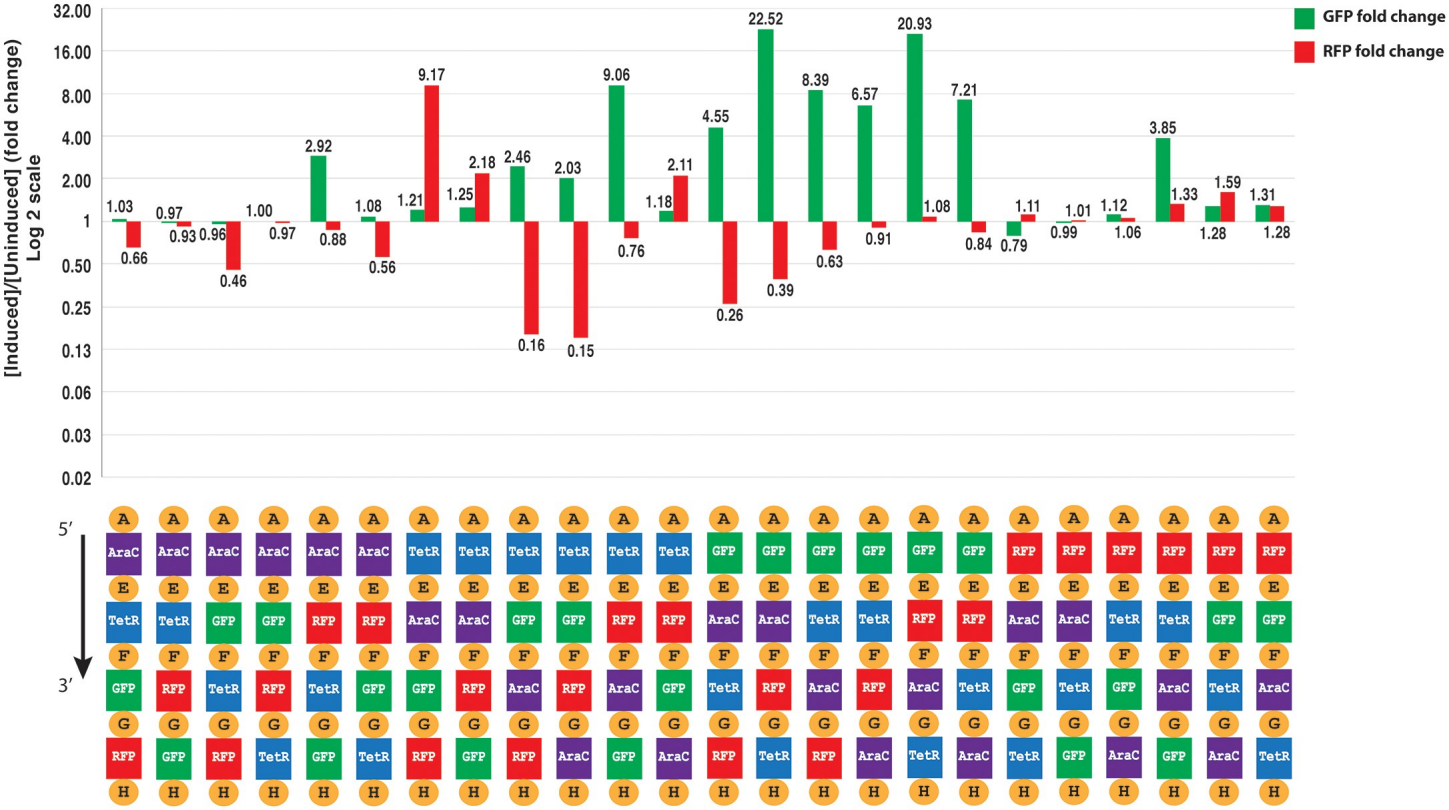
Comparison of GFP and RFP fold changes upon induction with L-arabinose of the 24-inverter permutation with no insulators. Most circuit permutations fail to provide anything close to the anticipated behavior, and even closely related circuits can yield radically different behavior.

As expected, the behavior of the uninsulated circuit is quite sensitive to local DNA context: Fig 3 shows the GFP and RFP fold changes upon induction with L-arabinose. Most permutations of the circuit fail to provide anything close to the anticipated behavior, showing significant device-to-device variation. Many do not even qualitatively act as an inverter: 8 out of 24 devices show almost no change in either their GFP or RFP expression levels on induction, and while GFP expression levels either remain unchanged or increase in almost all devices, in 8 of the 24 devices RFP expression levels increase rather than decrease upon arabinose induction. Any patterns are weak at best, with even closely related circuits yielding radically different behavior. For example, one of the best performing circuits (GFP-AraC-TetR-RFP) is only a single swap of transcription units away from the worst (TetR-AraC-GFP-RFP), dropping GFP induction by more than 3-fold and shifting RFP nearly 40-fold from significant repression to strong induction. Our test circuit thus appears quite strongly impacted indeed by differences in genetic context.

**Fig 4:**
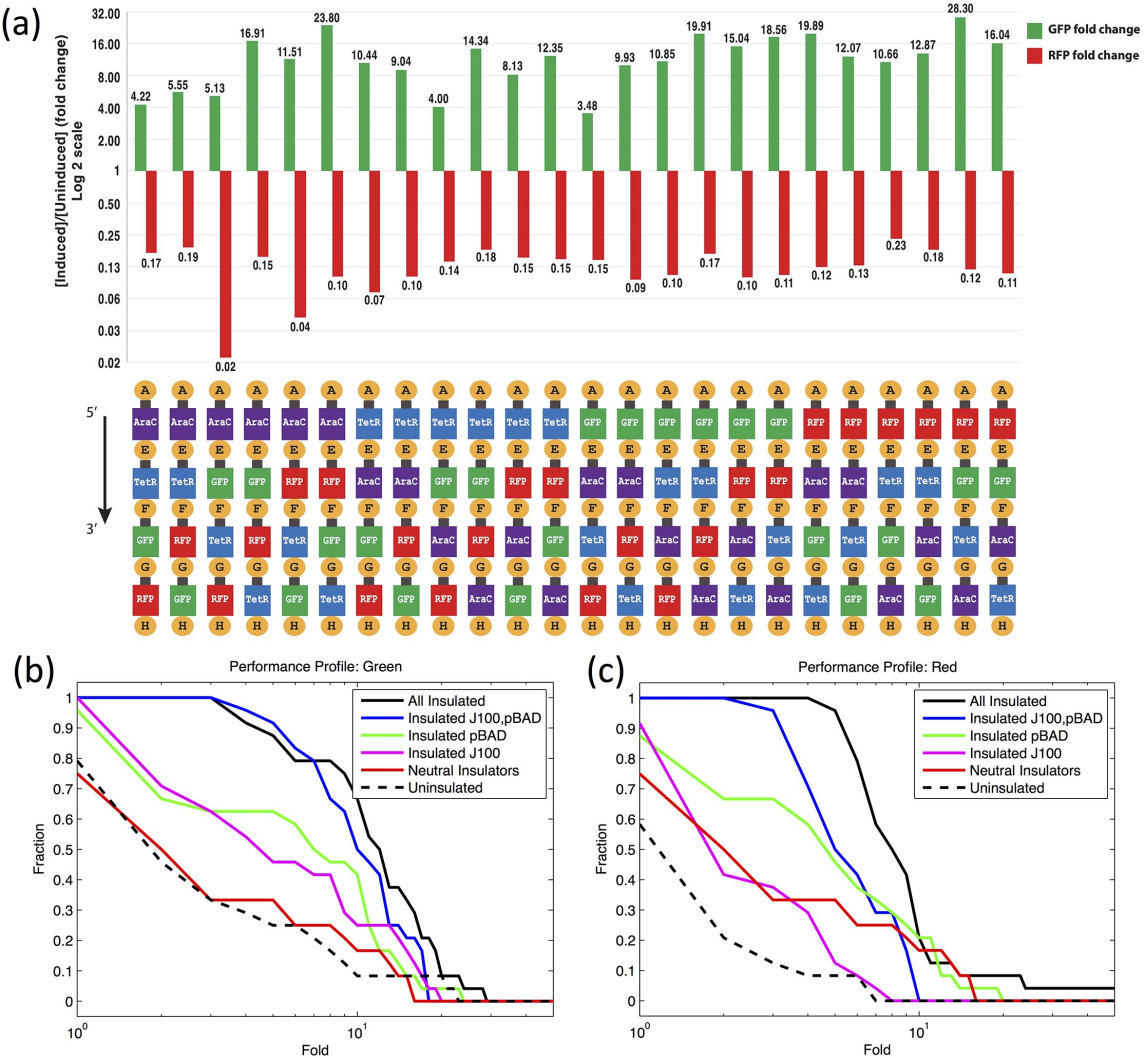
DIS insulation removes order dependence and improves device performance. (a) Comparison of GFP and RFP fold changes upon induction with L-arabinose of the 24-inverter permutation set with DIS spacers. Insulation is denoted by a smaller gray box positioned between upstream MoClo fusion site and colored box denoting a transcriptional unit. (b) and (c) summarize the performance of permutation sets with various levels of insulation, by showing the fraction of devices with at least a certain level of induction (e.g., only 46% of uninsulated devices—11 of 24—had 2-fold or better GFP induction). Both the performance of individual circuits and the number of circuits that perform well increases with each additional promoter that is insulated with DIS.

Insulation with DIS-generated spacers markedly improves both the strength and regularity of performance: Fig 4(a) shows the GFP and RFP fold changes of the fully insulated circuit upon induction with L-arabinose. Both strength and homogeneity of induction response is greatly improved, with every single permutation providing at least a 3-fold induction of GFP expression and at least a 4-fold repression of RFP expression. Order dependence is largely eliminated: qualitatively, every permutation is providing the same functionality, and while there is certainly still quantitative variation in performance, the geometric standard deviation of induction response is only 1.8-fold for GFP and 1.7-fold for RFP.

The in-context selection of DIS does indeed appear to be important for obtaining this effective insulation: randomly-generated spacers fail to effectively insulate from local DNA context, yielding performance nearly as bad as if there were no insulation at all (S6 Fig). We also find that each individual promoter’s insulation is significant, as performance improves incrementally with each promoter insulated: Fig 4(b) and 4(c) summarize the performance of all of the permutation sets, by showing the fraction of devices with at least a certain level of induction: better performance moves the curve both upward (more devices responding to induction) and rightward (stronger response to induction). Details are provided in S7–S9 Figs. Using DIS for insulating just J23100 or pBAD provide, respectively, a 2.5-fold and 1.9-fold mean improvement in induction ratios over uninsulated performance, insulating both J23100 and pBAD provides a 4.3-fold improvement, and insulating all three provides a 5.8-fold improvement. In summary: DIS appears to provide a simple and highly effective means of mitigating dependence on local DNA context and improving the expected performance of biological circuits.

## Discussion

If promoters are to be used as part of complex genetic designs, it is unavoidable that their upstream genetic context will change, since they may be placed after any number of other genetic constructs. Even small variations in the DNA sequence at the 5’ boundary of a promoter can greatly alter its behavior and cause design failures, and no method has yet been identified for reliably predicting the effect of upstream sequence on promoter behavior [12, 13, 16]. This problem is likely worsened by the fact that many of the promoters used in creating engineered genetic constructs are “minimal” constructs whose sequence includes little or nothing outside of whichever regions are known significant for a particular promoter (e.g., binding sites for transcriptional regulators or the transcription initiation complex). Accordingly, our DIS approach to insulating promoters from upstream genetic constructs is based on screening a promoter with many randomly generated spacers to identify those spacers that do produce the precise desired level of expression. We have demonstrated that this method can be effectively applied to insulate constitutive, inducible and repressible promoters, and that such insulation both regularizes and improves the performance of an “inverter” test circuit. Moreover, DIS can be implemented quickly and inexpensively with standard labware and is also highly susceptible to automation.

Some potential challenges in generalization of this approach remain for future investigation. For example, we note that the degree of variation observed in a collection of random insulators differs significantly between different promoters (Fig 1): it is thus possible (though it seems unlikely) that a distribution of randomly insulated promoters will not contain any samples sufficiently close to the required expression behavior, in which case it would be necessary to change the promoter and/or RBS components. Similarly, there is not expected to be significant interaction between the insulator and components downstream of the 3’ end of the promoter, but we have not actually validated this experimentally. Likewise, our method is predicted, but not verified, to be just as applicable to divergent and convergent promoter elements as we have shown it to be for individual promoters and for promoters in sequential transcriptional units. Lastly, the 36 nt length of our spacers was chosen to balance length with feasibility of the method for generating randomized libraries, but other lengths are likely to provide effective spacers as well: reducing length is likely to degrade the efficacy of insulation, while increasing length is likely to improve efficacy of insulation up to some point of diminishing returns.

Another important question for future work is to determine how broad a class of DNA context effects can actually be insulated against by means of this approach. The promoters tested in this paper have mechanisms that are prototypical and representative of a large class of commonly used genetic components, so it appears likely that the method we have presented will effectively insulate against a wide range of transcriptional/translational components. At the same time, there are clearly limits to the efficacy of spacers: for example, spacers provide no mechanism to protect against side effects from nearby epigenetic modification or sequence editing.

We also note that our screening method is defined in terms of a reference device to screen candidate insulators: in the absence of a defined reference that is desired as a target, our approach might instead be used for “tuning” to select a desired expression level from a given promoter or to adjust and optimize the performance of engineered biological systems. Likewise, although we have focused on bacterial promoters, there is nothing to prevent this technique from being applied to promoters in other organisms or to other components that prove to be sensitive to local genetic context.

Finally, many widely used bacterial promoters are either known to or likely to suffer from the upstream genetic context issues that we have addressed in this paper. We thus expect that it will be valuable for the synthetic biology community to begin applying our method to develop and characterize insulated variants of a wide variety of bacterial promoters. Furthermore, in many cases we expect that it will be useful to prepare not just one but multiple variants of an insulated promoter, as is readily supported by our method, so that if a promoter is used multiple times in a design, different insulators can be used in each case in order to minimize undesired homology. Developing such insulated promoter libraries will enable faster, simpler, and more reliable use of those promoters in new biological engineering efforts in both academia and industry.

## Materials and methods

A summary of key method information follows; additional details may be found in Supporting Information S1 File.

### 24 inverter set assembly

Basic parts (promoter, RBS, CDS, terminator) from the CIDAR-MoClo part collection [16] were assembled into transcriptional units (Level 1 MoClo parts) using an optimized MoClo DNA assembly standard [18]. Transcriptional units were subsequently assembled via a Level 2 MoClo assembly reaction into inverters. Insulated promoters were ordered as MoClo assembly compatible gblocks from Integrated DNA Technologies (Coralville, IA, USA). Spacer sequence was inserted 5’ of the promoter between promoter and upstream MoClo fusion site.

### Randomized 36 nt spacer generation and screening

Randomized 36 nt insulated promoter libraries were generated by performing an inverse PCR reaction using divergent primers to the promoter to be insulated. A phosphate group and 18N extension was added to the 5’ end of both forward and reverse primer. Overnight digestion of PCR product with DpnI (New England BioLabs, Ipswich, MA, USA) was performed to remove traces of PCR template. PCR product was purified using a GenCatch PCR Purification Kit (Epoch Life Science Inc., Missouri City, TX, USA). PCR product was ligated in an overnight 16°C blunt end ligation using T4 DNA ligase (New England BioLabs, Ipswich, MA, USA). The purified PCR product was ligated and transformed into *E. coli*. Expression of samples from the insulated library was measured by flow cytometry and compared against a reference device.

Spacers for constitutive promoter J23100 were selected by screening candidates from an insulated GFP expression cassette library of J23100_E (12.5X lower expression than J23100_A). Spacers for inducible and repressible promoters pBAD and pTet were obtained by screening insulated expression cassettes of pBAD_F (6.5X lower expression than pBAD_A and pTet_A, respectively. To ensure selected spacers did not disrupt transcription factor binding, inducible and repressible promoters pBAD and pTet were screened in their final context with and without induction. Candidate insulated devices that failed to respond strongly to induction were eliminated.

### Selected insulating sequences

The 36 nt spacers ultimately selected to serve as insulators had the following sequences:

- J23100: CCTGCAGTATTCATTTTCAGCTTACGGAAGGTAGAT
- pBAD (first): CGTTGTTCGCGACCCTGTTCTGAGGCAATGTTGGGC
- pBAD (second): TAGCACGCTTTTCTGCGTATGGTTGGTCAGCTTCTA
- pTet: CGGAGAGAATACATAGTAAAGAAACCTCACTGTTGT

Additional information related to these sequences may be found in Supporting Information S1 File.

### Bacterial strains and growth conditions

Transformations were performed using 3*μ*L of MoClo reaction mixture into 12-25 *μ*L of Alpha Select Gold Efficiency chemically competent cells (Bioline USA Inc., Taunton, MA, USA) following the manufacturer’s protocol. Cells were grown overnight at 37°C on MoClo assembly level-appropriate antibiotic selective LB agar supplemented with 80 *μ*L of 20mg/*μ*L 5-bromo-4-chloro-3-indolyl *β*-D-galactopyranoside (X-GAL) and 100 *μ*L of 0.1M isopropyl-*β*-D-thiogalactopyranoside (IPTG) (Zymo Research Corp., Irvine, CA USA) for blue-white screening.

### Plasmid isolation, construct size and sequence analysis

Plasmids were purified using the QIAprep Spin Miniprep kit (Qiagen) following the manufacturers’ protocols. Inverters were checked for correct size by performing colony PCRs on MoClo transformation colonies using 2X Taq Mastermix (New England BioLabs, Ipswich, MA, USA) and VF (5’-tgccacctgacgtctaagaa) and VR (5’-attaccgcctttgagtgagc) primers. Sequences were analyzed using Benchling (San Francisco, CA, USA).

### Flow cytometry

All fluorescent expression devices were characterized using a BD LSRFortessa SORP flow cytometer. RFP fluorescence was measured using a solid-state Coherent Sapphire 561 nm laser at 100 mw strength with a PE-Texas Red 610/20 filter. GFP fluorescence was measured using a solid-state Coherent Sapphire 488 nm laser at 200 mw strength with a FITC 530/30 filter. LB agar (Sigma-Aldrich, St. Louis, MO, USA) plates containing the appropriate antibiotic were inoculated with Alpha-Select Gold Efficiency *E. coli* cells (Bioline) containing the confirmed plasmid construct/clone from insulated promoter cassette library of interest. Colonies were grown in 200 *μ*L LB broth (Sigma-Aldrich) with the appropriate antibiotic in sterile 96-well, deep well plates (BioExpress, Kaysville, UT, USA) in triplicate (for confirmed plasmid construct) or singly (for insulated promoter expression cassette library) for 14 hours at 37°C shaking at 300 rpm. Cells were diluted into phosphate buffered saline (BioExpress) in 96-well round bottom plates before measurement using a high-throughput sampler (HTS).

### Statistical analysis

Flow cytometry data was converted from arbitrary units to compensated MEFL (Molecules of Equivalent Fluorescein) using the TASBE characterization method [19, 20]. An affine compensation matrix is computed from single color, dual color, and blank controls: RFP (red) alone (J23104:BCD2:E1010m:B0015 in the MoClo Level 1 destination vector DVL1_AE, abbreviated as pJ04B2Rm_AE), GFP (green) alone (J23104:BCD2:E0040m:B0015 in the MoClo Level 1 destination vector DVL1_AE, abbreviated as pJ04B2Gm_AE, the dual red-green color control (J23104:BCD2:E1010m:B0015:J23104:BCD2:E0040m:B0015 in the MoClo destination vector DVL2_AF, abbreviated as pJ04B2Rm:J04B2Gm_AF) and untransformed Alpha Select *E. coli* cells (Bioline, Tauton, MA, USA), respectively. FITC channel measurements (for GFP) are calibrated to MEFL using SpheroTech RCP-30-5-A beads.

## Supporting information

S1 File Additional method details. Additional information about methods employed, including all DNA sequence information.
(PDF)

S1 Fig Designed insulators. Variations in promoter expression amongst a collection of nine 36 nt designed neutral DNA spacer sequences. A preliminary test of the variable effect of random insulators on expression was performed with 36 nt spacer sequences designed by screening random DNA base composition resembling the intergenic bacterial DNA base composition for tandem promoter elements [21] and absence of secondary structures [22, 23] that could interfere with gene expression and promoter consensus sequences. Nine such spacers were inserted at the 5’ end of a J23100-RFP expression cassette, and fluorescent expression from each measured in triplicate. Error bars show +/- two std.dev. of mean fluorescence. While RFP expression levels from five of the spacers were statistically indistinguishable (starred bars), the remainder showed a significant variation in expression levels from spacer to spacer.
(PDF)

S2 Fig Upstream impact on promoters variations in promoter expression due to variations in the 4 bp MoClo assembly DNA sequence at the 5’ junction of promoters. Constitutive expression from (a) J23100 promoter, (b) pBAD inducible promoter, and (c) pTet repressible promoter, when downstream of MoClo A, E, F, and G sites. Error bars show +/- two std.dev. of mean fluorescence. The J23100 and pBAD promoters both show a significant difference in expression with different fusion sites, with more than a 10-fold difference in expression between the highest and lowest expression levels, while pTet expression did not vary significantly.
(PDF)

S3 Fig Insulator length comparison. Variations in promoter expression with length of designed neutral DNA spacer sequences. A preliminary test of the effect of insulator length on expression was performed with spacer sequences designed to several lengths (12 nt, 24 nt, and 36 nt) by screening random DNA base composition resembling the intergenic bacterial DNA base composition for tandem promoter elements [21] and absence of secondary structures [22, 23] that could interfere with gene expression and promoter consensus sequences. Two such spacers were inserted at the 5’ end of four different promoter-RFP expression cassettes, making eight combinations per spacer length, and fluorescent expression from each measured in triplicate. Error bars show +/- two std.dev. of mean fluorescence. While major variations in expression level between spacer pairs for a promoter were observed for 12 nt and 24 nt, only relatively low variation between pairs was observed for the 36 nt, suggesting this length may be sufficient to insulate promoters from changes in upstream sequence.
(PDF)

S4 Fig pBAD insulation ± data. Distribution of expression levels for induced and uninduced expression levels from spacer screen for pBAD, corresponding with fold-change distributions reported in Fig 1(c).
(PDF)

S5 Fig pTet insulation ± data. Distribution of expression levels for induced and uninduced expression levels from spacer screen for pTet, corresponding with fold-change distributions reported in Fig 1(d).
(PDF)

S6 Fig Designed insulators. 24-inverter library insulated using random spacers. Comparison of GFP and RFP fold changes upon induction with L-arabinose of the 24-inverter permutation set in which each transcriptional unit is insulated with a different spacer from S1 Fig (INS-1 for J23100, INS-2 and INS-3 for the two pBADs, and INS-5 for pTet.). Insulation is denoted by a smaller gray box positioned between upstream MoClo fusion site and colored box denoting a transcriptional unit. Performance remains highly unreliable, indicating that it is problematic to generate spacers without consideration of their usage context.
(PDF)

S7 Fig Insulated J23100 inverter. 24-inverter library with insulated J23100 promoter. Comparison of GFP and RFP fold changes upon induction with L-arabinose of the 24-inverter permutation set in which only the J23100 promoter has been insulated with a DIS spacer. Insulation is denoted by a smaller gray box positioned between upstream MoClo fusion site and colored box denoting a transcriptional unit. Performance improves over uninsulated inverters, but is not as good as inverters with more insulators.
(PDF)

S8 Fig Insulated pBAD inverter. 24-inverter library with insulated pBAD promoters. Comparison of GFP and RFP fold changes upon induction with L-arabinose of the 24-inverter permutation set in which only the two pBAD promoters have been insulated, each with a different DIS spacer. Insulation is denoted by a smaller gray box positioned between upstream MoClo fusion site and colored box denoting a transcriptional unit. Performance improves over uninsulated inverters, but is not as good as inverters with more insulators.
(PDF)

S9 Fig Insulated J23100 and pBAD inverter. 24-inverter library with insulated J23100 and pBAD promoters. Comparison of GFP and RFP fold changes upon induction with L-arabinose of the 24-inverter permutation set in which both the J23100 and two pBAD promoters have been insulated with a DIS spacer. Insulation is denoted by a smaller gray box positioned between upstream MoClo fusion site and colored box denoting a transcriptional unit. Performance improves over a single insulator inverter, but is not as good as insulating all promoters, particularly for change in RFP expression levels.
(PDF)

S1 Dataset Figure data. All data underlying the findings described herein.
(ZIP)
