# Supplementary material for: Reducing DNA context dependence in bacterial promoters
Source: PLoS One. 2017 Apr 19;12(4):e0176013. doi: 10.1371/journal.pone.0176013 (PMC5396932; doi:10.1371/journal.pone.0176013)
Supplement: S1 File — Additional information about methods employed, including all DNA sequence information. (PDF) [file pone.0176013.s001.pdf]

# Reducing DNA Context Dependence in Bacterial Promoters
# Supporting Information S1 File: Additional Method Details

Swati B. Carr, Jacob Beal, Douglas M Densmore

## 1 Materials

### 1.1 Media

The media used for growing the bacteria strains in this study are shown in Table 1:

| Medium | Vendor | Used for |
|---|---|---|
| Luria Bertani (LB) Broth (Lennox) | Sigma-Aldrich | General growth in liquid media |
| LB Broth with Agar (Lennox) | Sigma-Aldrich | General growth in solid media |
| SOC Broth | Sigma-Aldrich | Post-transformation recovery |
| L-Arabinose | Sigma-Aldrich | Small molecule inducer |

Table 1: Bacterial growth media used in this study

All media were autoclaved (121C for 15 minutes) or filter-sterilized (0.22 $\mu$m filter) prior to use.

### 1.2 Bacterial strains

All transformation and flow cytometry assays were performed in Alpha Select Gold Efficiency *E. coli* cells (Bioline USA Inc., Tauton, MA, USA) (Genotype: F- *deo*R *end*A1 *recA*1 *rel*A1 *gyr*A96 *hsd*R17(rk-, mk+) *sup*E44 thi-1 *pho*A $\Delta$(lacZYA-argF)U169 $\phi$80*lac*Z$\Delta$M15 $\lambda$-). Bacterial strains were streaked for single colonies from glycerol stocks onto solid agar media and grown overnight at 37°. Liquid cultures (see Section 3) were inoculated with single bacterial colonies and grown overnight at 37° with shaking (300 rpm).

### 1.3 Antibiotics

The final (1x) concentrations of antibiotics used for selective growth of *E. coli* in both solid and liquid media in this study are listed in Table 2:

| Antibiotic | Final Concentration | Vendor |
|---|---|---|
| Ampicillin | 100 $\mu$g/mL | Zymo Research |
| Kanamycin | 35 $\mu$g/mL | Zymo Research |
| Chloramphenicol | 25 $\mu$g/mL | Zymo Research |

Table 2: Antibiotics used in this study.

Relevant antibiotics were added to all bacterial growth media in 1x concentration.

### 1.4 Kits

Commercially available kits, used in this study as per manufacturer's protocols are listed in Table 3. Miniprep and maxiprep kits were used to purify DNA for sequence verification or for use in downstream DNA assembly reactions. PCR cleanup kits were used to remove PCR reagents and oligonucleotide primers from reactions before using in downstream DNA assembly steps.

| Kit | Company | Used for |
|---|---|---|
| QIAprep Spin Miniprep Kit | Qiagen | Purification of plasmid DNA |
| GenCatch Plasmid DNA Mini-Prep Kit | Epoch Life Science | Purification of plasmid DNA |
| NucleoBond Xtra Midi Plus | Machery-Nagel | Purification of plasmid DNA |
| QIAquick PCR Purification Kit | Qiagen | Purification of PCR product |
| GenCatch PCR Cleanup Kit | Epoch Life Science | Purification of PCR product |
| QIAquick Gel Extraction Kit | Qiagen | Purification of DNA from agarose gel |
| GenCatch Gel Extraction Kit | Epoch Life Science | Purification of DNA from agarose gel |

Table 3: Molecular kits used in this study.

## 1.5 Enzymes

Enzymes used in this study are described in Table 4. All enzymes were purchased either from New England BioLabs (Ipswich, MA, USA) or Promega Corp. (Madison, WI, USA). BsaI was used to construct Level 1 genetic circuits from basic (Level 0) parts. BbsI was used to construct Level 2 MoClo circuits from Level 1 circuits. Restriction digests were performed to verify genetic circuit size or to move a circuit into a different vector backbone. DpnI was used in the creation of insulated promoter libraries as described in Section 2.3.1.

| Enzyme | Vendor | Application |
|---|---|---|
| BsaI | New England BioLabs | MoClo DNA Assembly |
| BbsI | New England BioLabs | MoClo DNA Assembly |
| T4 Ligase | Promega | MoClo DNA Assembly |
| T4 Ligase | New England BioLabs | Ligation |
| EcoRI | New England BioLabs | Restriction Digestion |
| XbaI | New England BioLabs | Restriction Digestion |
| SpeI | New England BioLabs | Restriction Digestion |
| PstI | New England BioLabs | Restriction Digestion |
| DpnI | New England BioLabs | Removing PCR template DNA |

Table 4: Enzymes used in this study.

## 1.6 DNA parts and primers

The DNA parts listed in Table 5 were taken from the CIDAR Inventory of Composable Elements (ICE) Registry [1] and are also available from Addgene [2]. These parts were used in the assembly of higher level circuits in this study, as described below. Rationally insulated promoter parts and promoter parts insulated using our new custom DNA spacer generation and screening method were synthesized as gblocks from Integrated DNA Technologies (Coralville, Iowa, USA). Rationally and empirically (using new screening technique) insulated promoter parts that were used to create Level 1 MoClo circuits and Level 2 inverter devices are listed in Tables 6, 7, and 8 (randional design) and Table 9 (empirical).

All primers for sequencing and creating randomized insulated promoter libraries are listed in Table 10 and Table 11, respectively. Primers VF and/or VR were used to sequence all intermediate inverter MoClo

Level 1 circuits and to run colony PCRs (Section 2.4). Full inverters were sequence-verified with sequencing primers designed to AraC, TetR, GFP and RFP CDSs as listed in Table 10.

| **Basic DNA part name** | **Part Type** | **CIDAR-ICE ID** | **Addgene ID** |
|---|---|---|---|
| J23100_AB | constitutive promoter | CIDAR-490 | 65980 |
| J23100_EB | constitutive promoter | CIDAR-491 | 65981 |
| J23100_FB | constitutive promoter | CIDAR-492 | 65982 |
| J23100_GB | constitutive promoter | CIDAR-493 | 65983 |
| pBAD_AB | inducible promoter | CIDAR-526 | 66016 |
| pBAD_EB | inducible promoter | CIDAR-527 | 66017 |
| pBAD_FB | inducible promoter | CIDAR-528 | 66018 |
| pBAD_GB | inducible promoter | CIDAR-529 | 66019 |
| pTet_AB | repressible promoter | CIDAR-518 | 66008 |
| pTet_EB | repressible promoter | CIDAR-519 | 66009 |
| pTet_FB | repressible promoter | CIDAR-520 | 66010 |
| pTet_GB | repressible promoter | CIDAR-521 | 66011 |
| B0030m_BC | RBS/BCD | | 74394 |
| B0032m_BC | RBS/BCD | CIDAR-530 | 66020 |
| B0034m1_BC | RBS/BCD | CIDAR-531 | 66022 |
| BCD12_BC | RBS/BCD | CIDAR-533 | 66023 |
| E0040m_CD (GFP) | CDS | CIDAR-542 | 66032 |
| E1010m_CD (RFP) | CDS | CIDAR-543 | 66033 |
| C0040_CD (TetR) | CDS | CIDAR-537 | 66027 |
| C0080_CD (AraC) | CDS | CIDAR-539 | 66029 |
| B0015_DE | Double terminator | CIDAR-545 | 66035 |
| B0015_DF | Double terminator | CIDAR-546 | 66036 |
| B0015_DG | Double terminator | CIDAR-547 | 66037 |
| B0015_DH | Double terminator | CIDAR-548 | 66038 |
| DVLK_AE | destination vector | CIDAR-577 | 66067 |
| DVLK_EF | destination vector | CIDAR-578 | 66068 |
| DVLK_FG | destination vector | CIDAR-579 | 66069 |
| DVLK_GH | destination vector | CIDAR-580 | 66070 |
| DVLA_AH | destination vector | CIDAR-553 | 66043 |
| DVLA_AF | destination vector | CIDAR-551 | 66041 |
| DVLA_AG | destination vector | CIDAR-552 | 66042 |

Table 5: Basic MoClo DNA parts and vectors used in this study to construct higher level genetic circuits. All basic parts (except B0030m_BC) are archived and publicly available both through Addgene [2] and the CIDAR-ICE DNA Parts Registry [1]. Insulated promoters were derived basic promoter parts by inserting additional bases (12, 24 or 36 bp) to serve as DNA spacers between the promoter and its 5' flanking MoClo fusion site.

| Promoter | Sequence |
|---|---|
| 12INS1-J23100_E | gcttTCCCGAGTAAGTttgacggctagctcagtcctaggtacagtgctagctact |
| 12INS2-J23100_E | gcttCATATCTCCAAAttgacggctagctcagtcctaggtacagtgctagctact |
| 12INS1-J23101_E | gcttTCCCGAGTAAGTtttacagctagctcagtcctaggtattatgctagctact |
| 12INS2-J23101_E | gcttCATATCTCCAAAtttacagctagctcagtcctaggtattatgctagctact |
| 12INS1-J23104_E | gcttTCCCGAGTAAGTttgacagctagctcagtcctaggtattgtgctagctact |
| 12INS2-J23104_E | gcttCATATCTCCAAAttgacagctagctcagtcctaggtattgtgctagctact |
| 12INS1-pTet_E | gcttTCCCGAGTAAGTtccctatcagtgatagagattgacatccctatcagtgatagagatactgagcactact |
| 12INS2-pTet_E | gcttCATATCTCCAAAtccctatcagtgatagagattgacatccctatcagtgatagagatactgagcactact |

Table 6: Promoter parts insulated with 12 bp rationally designed spacers. Constitutive promoters J23100, J23100, J23104, and the repressible promoter pTet were insulated with 12 bp spacers. DNA spacers were placed between MoClo fusion site and immediately 5' of promoter. Sequence is read as MoClo 5' fusion site-Spacer-promoter-MoClo 3' fusion site. First four bases represent upstream MoClo fusion site. DNA spacer sequence is represented in capital letters. Promoter sequence follows spacer sequence. Final four bases represent the MoClo 3' fusion site immediately downstream of the promoter. The sequences listed below can also be found in the CIDAR Benchling parts collections.

| Promoter | Sequence |
|---|---|
| 24INS1-J23100_E | gcttCCATTGAGCCAGCTTCAGGGTTCAttgacggctagctcagtcctaggtacagtgctagctact |
| 24INS1-J23100_E | gcttGAGGTCCGTGCACCGTCAGAAGCCttgacggctagctcagtcctaggtacagtgctagctact |
| 24INS1-J23101_E | gcttCCATTGAGCCAGCTTCAGGGTTCAtttacagctagctcagtcctaggtattatgctagctact |
| 24INS1-J23101_E | gcttGAGGTCCGTGCACCGTCAGAAGCCtttacagctagctcagtcctaggtattatgctagctact |
| 24INS1-J23104_E | gcttCCATTGAGCCAGCTTCAGGGTTCAttgacagctagctcagtcctaggtattgtgctagctact |
| 24INS1-J23104_E | gcttGAGGTCCGTGCACCGTCAGAAGCCttgacagctagctcagtcctaggtattgtgctagctact |
| 24INS1-pTet_E | gcttCCATTGAGCCAGCTTCAGGGTTCAtccctatcagtgatagagattgacatccctatcagtgatagagatactgagcactact |
| 24INS1-pTet_E | gcttGAGGTCCGTGCACCGTCAGAAGCCtccctatcagtgatagagattgacatccctatcagtgatagagatactgagcactact |

Table 7: Promoter parts insulated with 24 bp spacers. Constitutive promoters J23100, J23100, J23104, and the repressible promoter pTet were insulated with 24 bp rationally designed spacers. DNA spacers were placed between MoClo fusion site and immediately 5' of promoter. Sequence is read as MoClo 5' fusion site-Spacer-promoter-MoClo 3' fusion site. First four bases represent upstream MoClo fusion site. DNA spacer sequence is represented in capital letters. Promoter sequence follows spacer sequence. Final four bases represent the MoClo 3' fusion site immediately downstream of the promoter. The sequences listed below can also be found in the CIDAR Benchling parts collections.

| Promoter | Sequence |
|---|---|
| 36INS1-J23100_E | gcttCCTGCAGTATTCATTTTCAGCTTACGGAAGGTAGATttgacggctagctcagt cctaggtacagtgctagctact |
| 36INS2-J23100_E | gcttAGCAGTTACTCAAGTGGTCAGGAGGCAGACGCAGCGttgacggctagctcagt cctaggtacagtgctagctact |
| 36INS1-J23100_A | ggagCCTGCAGTATTCATTTTCAGCTTACGGAAGGTAGATttgacggctagctcagt cctaggtacagtgctagctact |
| 36INS2-J23100_A | ggagAGCAGTTACTCAAGTGGTCAGGAGGCAGACGCAGCGttgacggctagctcagt cctaggtacagtgctagctact |
| 36INS3-J23100_A | ggagCCTTTTCACGAGTCGCGTACTCTCCTAACTATCAAGttgacggctagctcagt cctaggtacagtgctagctact |
| 36INS4-J23100_A | ggagTTAATCAGTGATAAGTTAAGATGCAACCAGAAGGCAttgacggctagctcagt cctaggtacagtgctagctact |
| 36INS5-J23100_A | ggagACCCGTACCGGAGCTCAATAACGGTGCGGCGTTTGGttgacggctagctcagt cctaggtacagtgctagctact |
| 36INS6-J23100_A | ggagAAGCGTTACAGCTCTTGTCCGTCCTAGGCAGCACTTttgacggctagctcagt cctaggtacagtgctagctact |
| 36INS7-J23100_A | ggagAGGGACGACCCGCGCACAGAATGTGTCGGGGTAACAttgacggctagctcagt cctaggtacagtgctagctact |
| 36INS8-J23100_A | ggagGGGTACAAAATTAAAAACACAGTGATGGAACCGTCCttgacggctagctcagt cctaggtacagtgctagctact |
| 36INS9-J23100_A | ggagCATCTAGTTGCGGAAAGTGTGTCACGAGTATCACGTttgacggctagctcagt cctaggtacagtgctagctact |
| 36INS10-J23100_A | ggagTAGGGGGCAAACATAACTCTCGTTCCGGGGAATGGCttgacggctagctcagt cctaggtacagtgctagctact |
| 36INS1-J23101_E | gcttCCTGCAGTATTCATTTTCAGCTTACGGAAGGTAGATtttacagctagctcagt cctaggtattatgctagctact |
| 36INS2-J23101_E | gcttAGCAGTTACTCAAGTGGTCAGGAGGCAGACGCAGCGtttacagctagctcagt cctaggtattatgctagctact |
| 36INS1-J23104_E | gcttCCTGCAGTATTCATTTTCAGCTTACGGAAGGTAGATttgacagctagctcagt cctaggtattgtgctagctact |
| 36INS2-J23104_E | gcttAGCAGTTACTCAAGTGGTCAGGAGGCAGACGCAGCGttgacagctagctcagt cctaggtattgtgctagctact |
| 36INS1-pTet_E | gcttCCTGCAGTATTCATTTTCAGCTTACGGAAGGTAGATtccctatcagtgatagagattgac atccctatcagtgatagagatactgagcactact |
| 36INS2-pTet_E | gcttAGCAGTTACTCAAGTGGTCAGGAGGCAGACGCAGCGtccctatcagtgatagagattgac atccctatcagtgatagagatactgagcactact |

Table 8: Promoter parts insulated with 36nt spacers. 36nt DNA spacers were placed between 5' boundary and upstream MoClo fusion site of promoters J23100, J23100, J23104, pBAD and pTet. First and final 4 bp represent 5' and 3' MoClo fusion sites. DNA spacer sequence is in capital letters. Promoter sequence follows spacer sequence. Final four bases represent the MoClo 3' fusion site immediately downstream of the promoter.

| Promoter | Length of DNA spacer | Addgene ID |
|---|---|---|
| 36INS-J23100_AB | 36 bp | 78666 |
| 36INS-J23100_EB | 36 bp | 78667 |
| 36INS-J23100_FB | 36 bp | 78668 |
| 36INS-J23100_GB | 36 bp | 78669 |
| 36INS1-pBAD_AB | 36 bp | 78670 |
| 36INS1-pBAD_EB | 36 bp | 78671 |
| 36INS1-pBAD_FB | 36 bp | 78672 |
| 36INS1-pBAD_GB | 36 bp | 78673 |
| 36INS2-pBAD_AB | 36 bp | 78674 |
| 36INS2-pBAD_EB | 36 bp | 78675 |
| 36INS2-pBAD_FB | 36 bp | 78676 |
| 36INS2-pBAD_GB | 36 bp | 78677 |
| 36INS-pTet_AB | 36 bp | 78678 |
| 36INS-pTet_EB | 36 bp | 78679 |
| 36INS-pTet_FB | 36 bp | 78680 |
| 36INS-pTet_GB | 36 bp | 78681 |

Table 9: List of 36nt insulated promoters used in this study. These promoters were created using DNA spacers selected using the presented screening methodology and are available through Addgene [2].

| Primer Name | Sequence |
|---|---|
| VF | tgccacctgacgtctaagaa |
| VR | attaccgcctttgagtgagc |
| AraC-seq-for | attcggagctgctggcgataaatct |
| AraC-seq-rev | tgatcgctgatgtactgacaagcct |
| TetR-seq-for | acgctaaaagttttagatgtgctttact |
| TetR-seq-rev | tgagtgcatataatgcattctctagtg |
| GFP-seq-for | acgtgctgaagtcaagtttgaaggt |
| GFP-seq-rev | tgccatgatgtatacattgtgtgagt |
| RFP-seq-for | aggtttcaaatgggaacgtgttatgaa |
| RFP-seq-rev | ggaagttggtaccacgcagtttaact |

Table 10: List of primers used for sequencing.

| Primer Name | Sequence | Function |
| --- | --- | --- |
| 36N-J00B12Gm-For | NNNNNNNNNNNNNNNNNN ttgacggctagctcagtcctaggtacagtgctagctactgggcc | J23100 library creation |
| 36N-J00B12Gm_A-Rev | NNNNNNNNNNNNNNNNNN ctccatgtcttccactagtctctagaagcggccgcgaattccag | J23100 library creation |
| 36N-J00B12Gm_E-Rev | NNNNNNNNNNNNNNNNNN aagcatgtcttccactagtctctagaagcggccgcgaattccag | J23100 library creation |
| 36N-J00B12Gm_F-Rev | NNNNNNNNNNNNNNNNNN agcgatgtcttccactagtctctagaagcggccgcgaattccag | J23100 library creation |
| 36N-J00B12Gm_G-Rev | NNNNNNNNNNNNNNNNNN ggcaatgtcttccactagtctctagaagcggccgcgaattccag | J23100 library creation |
| 36N-pBAD-For | NNNNNNNNNNNNNNNNNN acattgattatttgcacggcgtcacactttgctatgccatagca | pBAD library creation |
| 36N-DVL2_F-Rev | NNNNNNNNNNNNNNNNNN agcgtgagacccactagtctctagaagcggccgcgaattccaga | pBAD library creation |
| 36N-pTet-For | NNNNNNNNNNNNNNNNNN tccctatcagtgatagagattgacatccctatcagtgatagaga | pTet library creation |
| 36N-DVL2_A-Rev | NNNNNNNNNNNNNNNNNN ctcctgagacccactagtctctagaagcggccgcgaattccaga | pTet library creation |

Table 11: **List of primers used to generate insulated promoter libraries.**

# 2 Methods

## 2.1 DNA purification

Plasmid DNA was purified from target bacterial cells grown in a 5 mL overnight LB culture supplemented with the appropriate antibiotic using the QIAprep Spin Miniprep kit (Qiagen), GenCatch Plasmid DNA Mini-prep kit (Epoch Life Science) or NucleoBond Xtra Midi Plus kit (Macherey-Nagel) according to the manufacturers instructions. The plasmids were eluted in a final volume of 50 $\mu$L of GenCatch Elution Buffer (10 mM Tris-Cl, pH 8.5) for mini-prep protocols and in 300$\mu$L GenCatch Elution Buffer for midi-prep protocols.

## 2.2 Modular Cloning (MoClo) DNA assembly

An updated and optimized protocol for the single pot, multi-part, Type IIS restriction endonuclease-based Modular Cloning (MoClo) DNA assembly method first described in 2011 [3] was used to generate all MoClo Level 1 monocistronic inverter/NOT-gate circuits. All basic DNA parts (Table 5) were either obtained from the CIDAR-ICE registry [1, 4] or were purchased as gblocks from Integrated DNA Technologies (Coralville, Iowa, USA) with the addition of DNA spacers and 4 bp MoClo-format overhangs (fusion sites) for assembly into MoClo Level 1 circuits. Level 1 MoClo circuits were assembled into Level 2 MoClo circuits (inverters) by standard MoClo assembly as described here [4, 5].

### 2.2.1 MoClo Level 1 part assembly from basic DNA parts in MoClo format

A transcriptional unit (promoter::RBS::CDS::terminator) or Level 1 MoClo part can be constructed from basic/Level 0 MoClo parts by combining equimolar amounts (4-40 fmol) of each basic part into a Level 1 destination vector in a one pot restriction digestion-ligation reaction using BsaI and T4 DNA ligase enzyme (Table 4). Level 0 parts have overlapping fusion sites such that the 3' fusion site of the upstream part must match the 5' fusion site of the downstream part for accurate directional assembly. The destination vector fusion sites must match the 5' fusion site of the promoter and the 3' fusion site of the terminator respectively. Level 0 parts have either chloramphenicol or ampicillin resistance while Level 1 vectors have kanamycin resistance and use *lacZ*$\alpha$ blue-white selection.

#### 2.2.2 MoClo Level 2 part assembly from Level 1 MoClo parts

Level 2 MoClo parts (higher-level genetic devices comprising multiple MoClo monocistronic transcriptional units) can be made by combining equimolar amounts of Level 1 MoClo parts (individual transcriptional units) with an ampicillin-resistant Level 2 destination vector in a one pot, restriction digestion-ligation reaction using BbsI (also called BpiI) and T4 DNA ligase (Table 4). The 3'-flanking fusion site of the first transcriptional unit must overlap with the 5'-flanking fusion site of the second transcriptional unit etc., for accurate directional assembly. Level 2 MoClo reactions also use *lacZα* blue-white selection.

#### 2.2.3 Modular Cloning (MoClo) Reaction Parameters and reaction protocol:

Each of four DNA parts (Level 0 parts for Level 1 MoClo reaction, and 2-4 Level 1 MoClo parts for Level 2 MoClo reaction) along with destination vector (DVL_1 or DVL_2 for Level 1 and Level 2 MoClo reactions, respectively) with fusion sites appropriate to insert parts were diluted to 10-20 fmol concentrations and added in equimolar amounts to the reaction. DNA parts can be purified plasmid DNA, PCR fragment or synthesized double-stranded DNA. DNA parts were combined with 1x T4 DNA ligase buffer (Promega), 5 units of BsaI (New England Biolabs) for Level 1 MoClo reaction or 10 units for Level 2 MoClo reaction, along with 120 units of T4 DNA ligase enzyme (New England BioLabs). The reaction was brought up to a final volume of 10 $\mu$L. MoClo reactions were performed in an Eppendorf Mastercycler ep thermocycler (Eppendorf) using the following parameters: 25 cycles (37°C for 1.5 minutes, 16°C for 3 minutes), 50°C for 5 minutes and 80° for 10 minutes. Reactions were then held at either 4° or -20° until transformed.

### 2.3 Randomized spacer generation and screening

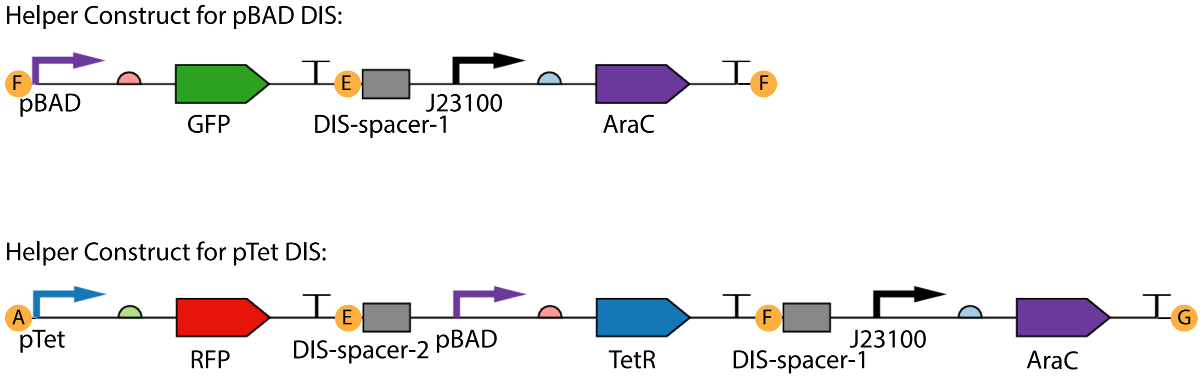


Figure 1: Helper constructs used for application of DIS to pBAD and pTet

#### 2.3.1 Randomized spacer generation

A 100% sequence-randomized, 36 bp insulated promoter library for a target MoClo promoter was generated by performing an inverse PCR reaction using divergent primers containing phosphorylated 18N (NNNNNNNNNNNNNNNNNN) 5' overhangs using either a constitutively expressed GFP expression cassette (J23100), an inducible GFP expression cassette (pBAD) or a repressible RFP expression cassette (pTet) as template (see Figure 1). PCR was followed by a blunt-end ligation followed by transformation and plating on LB agar with appropriate antibiotics.

To enable subsequent screening of insulated promoters (see Figure 2), the target promoter was assembled into a GFP-expression cassette and the resulting transcriptional unit (TU) was used as the PCR template.

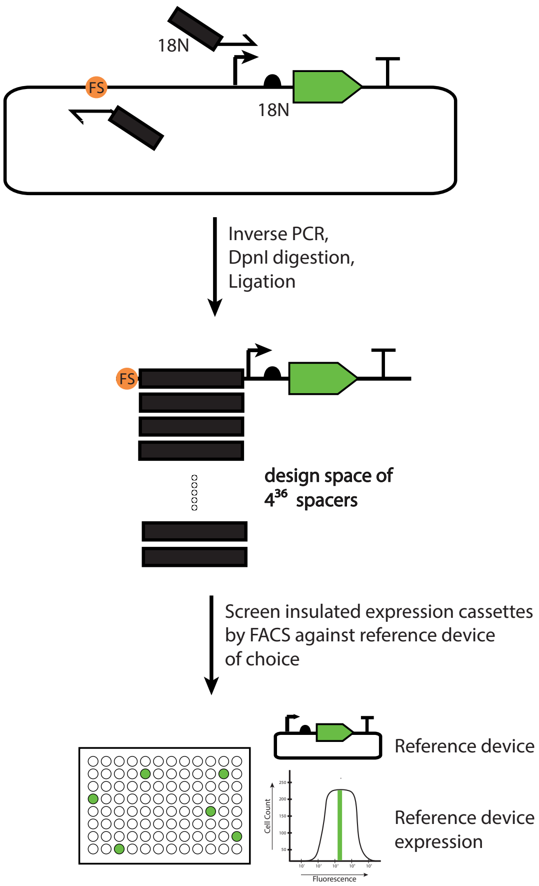


Figure 2: Divergent forward and reverse primers with 18N (NNNNNNNNNNNNNNNNNN) 5' primer extensions were used in an inverse PCR reaction using a plasmid containing the target genetic circuit as PCR template. The forward primer is designed to anneal to the promoter that is to be insulated. The reverse primer is designed to anneal to the MoClo fusion site immediately upstream of the promoter. The PCR reaction will result in a library of linearized plasmid DNA strands with the target promoter insulated. Overnight DpnI digestion removes residual template DNA. The linear plasmid PCR products are circularized in an overnight blunt-end ligation and then transformed and plated onto LB agar with appropriate antibiotics. Colonies from the library are grown overnight in liquid media, diluted before taking fluorescence measurements using a flow cytometer. The fluorescence expression of a reference device is used to screen the library of insulated expression cassettes for ones with the precise level of desired fluorescence expression. Plasmid DNA from the selected insulated cassettes is purified using commercial DNA purification kits and then sequenced. The DNA spacer sequence thus obtained is used to design new promoter parts for the final target device.

The uninsulated transcriptional unit plasmid DNA was eliminated by overnight DpnI digestion followed by overnight blunt-end ligation as shown in Figure 2.

The forward primer was designed to include (from 5' to 3') a 100% degenerate 18N sequence followed by the promoter and further downstream sequence up to 44 bases. The reverse primer was designed to include (from 5' to 3') a 100% degenerate 18N sequence, followed by the MoClo fusion site immediately upstream of promoter and further upstream sequence up to 44 bases. The primers were ordered phosphorylated and PAGE-purified from the manufacturer (Integrated DNA Technologies, Coralville, Iowa, USA). Eight 50$\mu$L PCR reactions were set up using High Fidelity (HF) Phusion polymerase (New England BioLabs, Ipswich, MA, USA). Reactions were set up as per the manufacturer's recommended protocol. The PCR products were pooled together. Template plasmid DNA was eliminated from the PCR product by overnight DpnI digestion of PCR product at 37° followed by heat inactivation of enzyme at 80° for 20 minutes. DpnI digestion was carried out as outlined in Section 2.3.2. The digested PCR product was purified (Table 3) and held at -20° until ligation.

### 2.3.2 Ligation and transformation

Blunt-end ligation was performed on the purified, digested and heat-inactivated PCR product at 16°C overnight in an Eppendorf Mastercycler ep thermocycler (Eppendorf). Ligated product was either held on ice or stored at -20° until ready to be transformed. The ligated PCR product contains the fully constructed insulated promoter cassette library. 5 $\mu$L of ligated insulated promoter expression cassette library was transformed into 25 $\mu$L of *E. coli* Alpha Select Gold Efficiency chemically competent *E. coli* cells (Bioline USA Inc., Tauton, MA, USA) as described in Section 2.5.

### 2.3.3 Randomized spacer screening

For tested promoters, spacer screening was performed using an upstream (5') MoClo fusion site that was suspected to strongly affect promoter expression. Colonies from the insulated promoter expression cassette library were grown overnight in LB broth with appropriate antibiotics. Fluorescence expression for colonies were tested by flow cytometry for either GFP (promoters J23100 and pBAD) or RFP (promoter pTet). For constitutive promoter J23100, insulated J23100 samples with GFP expression levels identical to the J23100-GFP_A cassette were selected from the tested samples as candidates for DNA spacers in target genetic devices. For promoter pBAD, insulated pBAD-GFP samples with GFP expression identical to that of pBAD-GFP_A in the induced state were selected as candidate DNA spacer sequences. For repressible promoter pTet, insulated pTet-RFP samples demonstrating RFP repression of >10x were selected as candidate spacer sequences.

## 2.4 Colony PCR

Fully assembled inverter devices were first checked for size by performing a colony PCR using standard PCR reagents and VF and VR primers (Table 10) to check the size of the cloned product after the MoClo Level 2 reaction (Section 2.2). PCR reaction was performed with an initial denaturation step at 95°C for 30 seconds, followed by 25 cycles each of 95°C for 20 seconds, 61°C for 20 seconds (primer annealing) and 68°C for 1 minute per kilobase of DNA in reaction (primer extension). After cycling, a final extension of 7 minutes at 68°C was performed. Reactions were held at 4°C until ready to be checked on a gel. All inverters were checked for size by electrophoresis through a 1% agarose gel using a 2-log ladder (New England BioLabs, Ipswich, MA, USA) as molecular size markers. Inverters that were the correct size were sequenced using sequencing primers designed to AraC, TetR, GFP and RFP CDSs (Table 10).

## 2.5 Transformation and selection

For transformation of MoClo reactions, 3 $\mu$L of the MoClo (Level 1 or Level 2) reaction mixture was combined with minimal handling with 7-20 $\mu$L of Alpha Select Gold Efficiency chemically competent *E. coli*

cells (Bioline USA Inc., Tauton, MA, USA) that had been thawed on ice. For transformation of ligation mixture from blunt-end ligation for randomized insulated cassette generation, 5 $\mu$L of the ligation mixture was combined with 25 $\mu$L of Alpha Select Gold Efficiency chemically competent *E. coli* cells (Bioline USA Inc., Tauton, MA, USA) that had been thawed on ice.

The mixture was allowed to incubate on ice for 2-15 minutes before being heat shocked in a 42° water bath for 30-45 seconds. The cells were recovered with 1 mL of SOC broth for 45-60 minutes at 37° shaking (300 rpm) before being plated on appropriate antibiotic selective LB agar plates supplemented with 80 $\mu$L of 20mg/$\mu$L 5-bromo-4-chloro-3-indolyl $\beta$-D-galactopyranoside (X-GAL) and 100 $\mu$L of 0.1M isopropyl-$\beta$-D-thiogalactopyranoside (IPTG) (Zymo Research Corp., Irvine, CA USA).

White colonies were selected from the overnight transformation plate and grown overnight at 37° shaking in LB broth medium with appropriate antibiotic. For MoClo reactions, plasmid DNA was purified using DNA purification using plasmid purification kits as per manufacturer's instructions (Table 3) and sent for DNA sequencing. For randomized spacer generation, selected colonies were screened by flow cytometry (see Section 3.2).

### 2.6 DNA sequencing

For purified plasmid DNA sequencing, a 10 $\mu$L reaction was prepared containing 150-200 ng of purified plasmid DNA, 1.67 pmol/$\mu$L sequencing primer (1 $\mu$L primer from a 10 $\mu$M stock) and deionized water to 10 $\mu$L. The spacer library was sequenced by submitting unpurified colony PCR samples and 10$\mu$M primer stock. All sequencing reactions were submitted to QuintaraBio for Sanger sequencing. Sequence analysis of trace files was performed using Benchling.

## 3 In vivo assay methods

### 3.1 Growth of bacterial cells

Single bacterial colonies were inoculated into culture tubes with LB broth with appropriate antibiotics. Inoculated cultures were grown 14-17 hours to a density of approximately 34 x $10^9$ cells per mL.

### 3.2 Fluorescence measurements by flow cytometry

All fluorescent expression devices were characterized using a BD LSRFortessa SORP flow cytometer. RFP fluorescence was measured using a solid-state Coherent Sapphire 561 nm laser at 100 mw strength with a PE-Texas Red 610/20 filter. GFP fluorescence was measured using a solid-state Coherent Sapphire 488 nm laser at 200 mw strength with a FITC 530/30 filter. LB agar (Sigma-Aldrich, St. Louis, MO, USA) plates containing the appropriate antibiotic were inoculated with Alpha-Select Gold Efficiency *E. coli* cells (Bioline) containing the confirmed plasmid construct/clone from insulated promoter cassette library of interest. Colonies were grown in 200 $\mu$L LB broth (Sigma-Aldrich) with the appropriate antibiotic in sterile 96-well, deep well plates (BioExpress, Kaysville, UT, USA) in triplicate (for confirmed plasmid construct) or singly (for insulated promoter expression cassette library) for 14 hours at 37°C shaking at 300 rpm. Cells were then diluted 100-fold into 200 $\mu$L of 1x sterile phosphate buffered saline (BioExpress) in 96-well round bottom plates before measurement using a high-throughput sampler (HTS).

### 3.3 Molecules of Equivalent Fluorescein (MEFL) conversion

Flow cytometry data was converted from arbitrary units to compensated MEFL (Molecules of Equivalent Fluorescein) using the TASBE characterization method [6, 7]. An affine compensation matrix is computed from single color, dual color, and blank controls: RFP (red) alone (J23104:BCD2:E1010m:B0015 in the MoClo Level 1 destination vector DVL1_AE, abbreviated as pJ04B2Rm_AE), GFP (green) alone (J23104:BCD2:E0040m:B0015 in the MoClo Level 1 destination vector DVL1_AE, abbreviated as pJ04B2Gm_AE,

the dual red-green color control (pJ04B2Rm:J04B2Gm) in the MoClo destination vector DVL2_AF, abbreviated as pJ04B2Rm:J04B2Gm_AF) and untransformed Alpha Select *E. coli* cells (Bioline, Tauton, MA, USA), respectively. FITC channel measurements (for GFP) are calibrated to MEFL using SpheroTech RCP-30-5-A beads.

# References


[1] Paige S, Iverson S, Heuckroth A, Carr S, Haddock T, Densmore D. The Boston University Center of Synthetic Biology (CoSBi) ICE Repository; Accessed, December 1, 2016. https://cidar.bu.edu/cosbi-registry/.

[2] Addgene. Addene Non-profit plasmid repository; 2016. Available from: `https://www.addgene.org/`.

[3] Weber E, Engler C, Gruetzner R, Werner S, Marillonnet S. A modular cloning system for standardized assembly of multigene constructs [Journal Article]. PLoS One. 2011;6(2):e16765. Available from: `http://www.ncbi.nlm.nih.gov/pubmed/21364738`.

[4] Haddock TL, Densmore DM, Appleton E, Carr S, Iverson S, De Freitas M, et al. BBF RFC 94: Type IIS Assembly for Bacterial Transcriptional Units: A Standardized Assembly Method for Building Bacterial Transcriptional Units Using the Type IIS Restriction Enzymes BsaI and BbsI; 2015.

[5] Iverson SV, Haddock TL, Beal J, Densmore DM. CIDAR MoClo: Improved MoClo Assembly Standard and New E. coli Part Library Enable Rapid Combinatorial Design for Synthetic and Traditional Biology [Journal Article]. ACS Synth Biol. 2016;5(1):99–103. Available from: `http://www.ncbi.nlm.nih.gov/pubmed/26479688`.

[6] Beal J, Weiss R, Yaman F, Davidsohn N, Adler A. A Method for Fast, High-Precision Characterization of Synthetic Biology Devices. MIT; 2012. MIT-CSAIL-TR-2012-008. Technical Report: MIT-CSAIL-TR-2012-008.

[7] Beal J. Bridging the gap: a roadmap to breaking the biological design barrier. Frontiers in bioengineering and biotechnology. 2015;2.
