# Supplementary figures and images for: Reducing DNA context dependence in bacterial promoters
Source: PLoS One. 2017 Apr 19;12(4):e0176013. doi: 10.1371/journal.pone.0176013 (PMC5396932; doi:10.1371/journal.pone.0176013)

RFP Expression in MEFLS

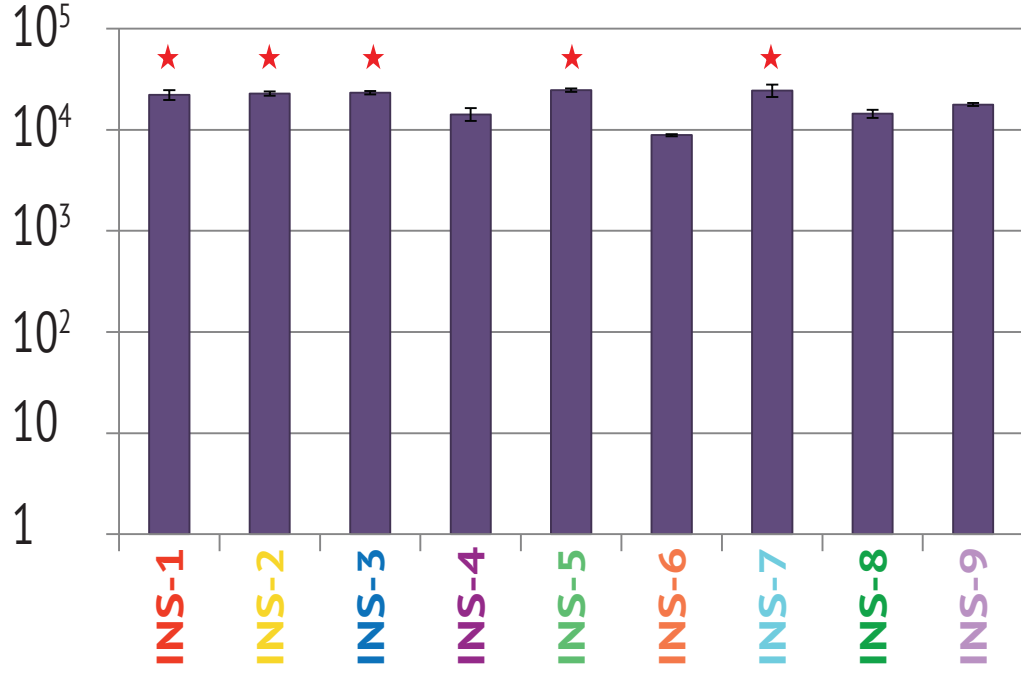

Supplement: S1 Fig — A preliminary test of the variable effect of random insulators on expression was performed with 36 nt spacer sequences designed by screening random DNA base composition resembling the intergenic bacterial DNA base composition for tandem promoter elements [21] and absence of secondary structures [22, 23] that could interfere with gene expression and promoter consensus sequences. Nine such spacers were inserted at the 5’ end of a J23100-RFP expression cassette, and fluorescent expression from each measured in triplicate. Error bars show +/- two std.dev. of mean fluorescence. While RFP expression levels from five of the spacers were statistically indistinguishable (starred bars), the remainder showed a significant variation in expression levels from spacer to spacer. (PDF) [file pone.0176013.s002.pdf]

(a)

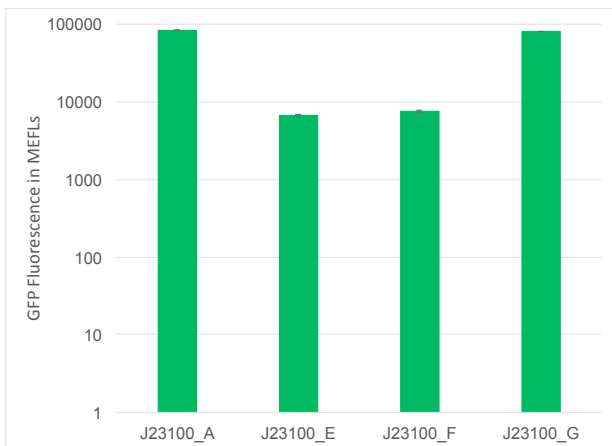

(b)

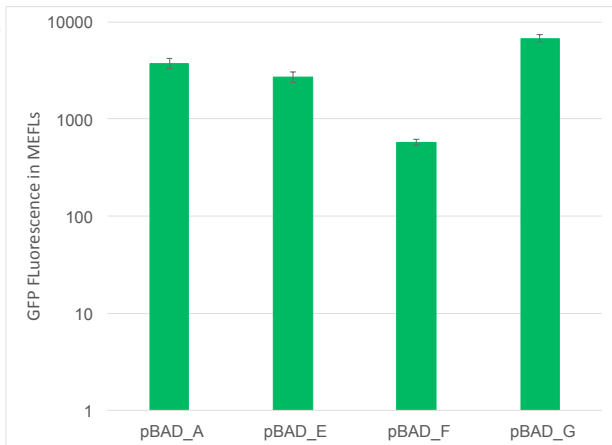

(c)

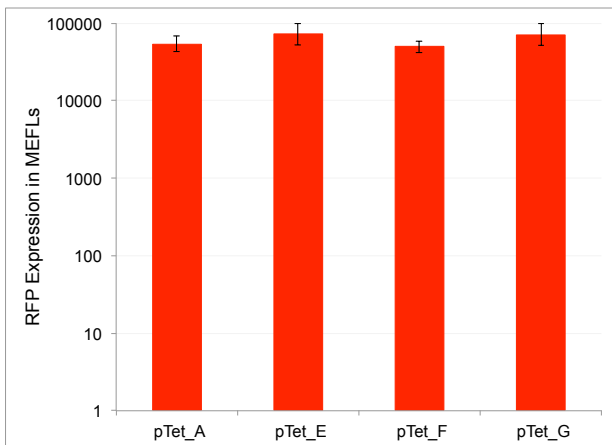

Supplement: S2 Fig — Constitutive expression from (a) J23100 promoter, (b) pBAD inducible promoter, and (c) pTet repressible promoter, when downstream of MoClo A, E, F, and G sites. Error bars show +/- two std.dev. of mean fluorescence. The J23100 and pBAD promoters both show a significant difference in expression with different fusion sites, with more than a 10-fold difference in expression between the highest and lowest expression levels, while pTet expression did not vary significantly. (PDF) [file pone.0176013.s003.pdf]

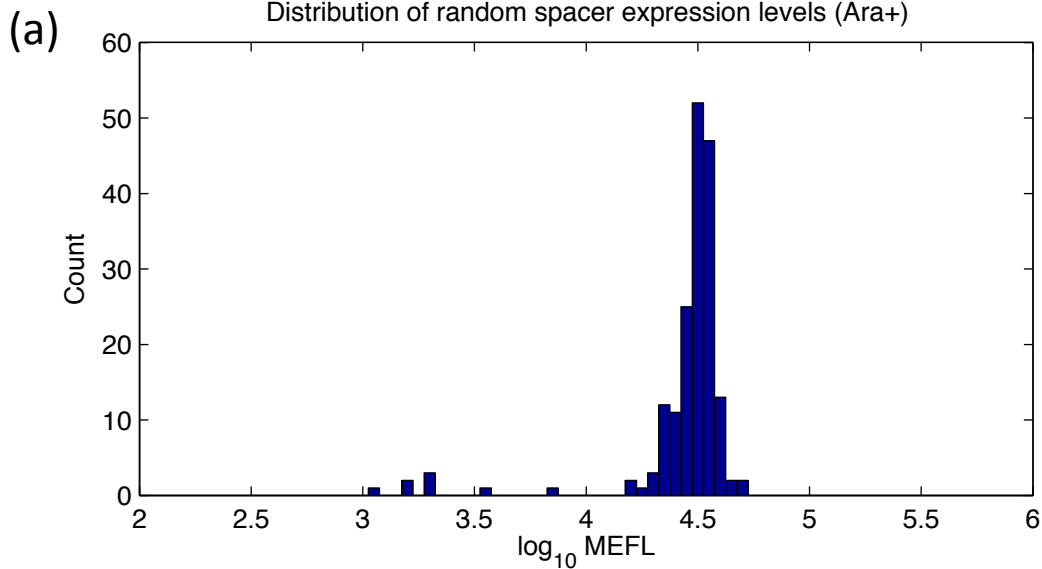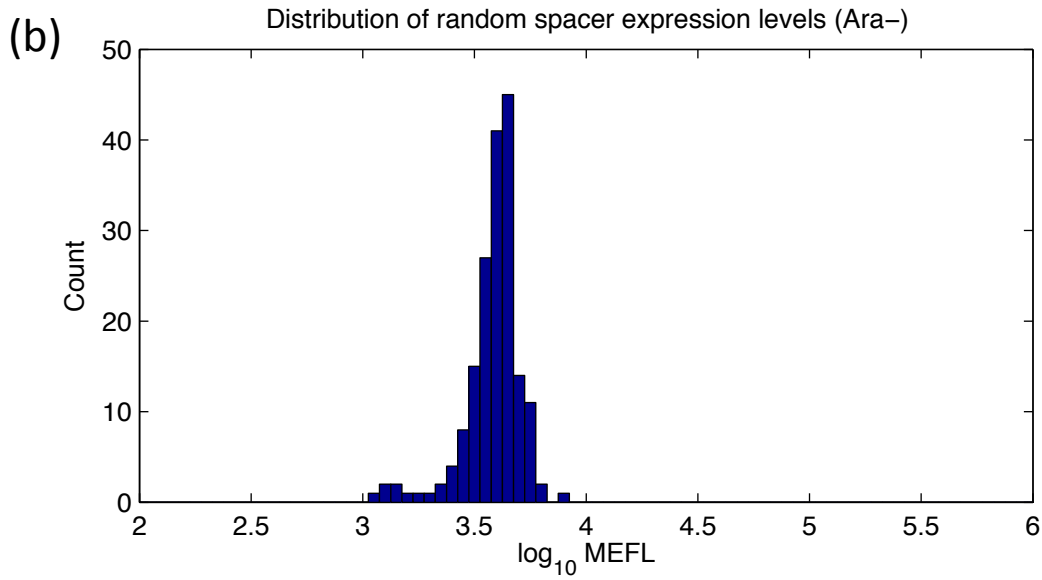

Supplement: S4 Fig — Distribution of expression levels for induced and uninduced expression levels from spacer screen for pBAD, corresponding with fold-change distributions reported in Fig 1(c). (PDF) [file pone.0176013.s005.pdf]

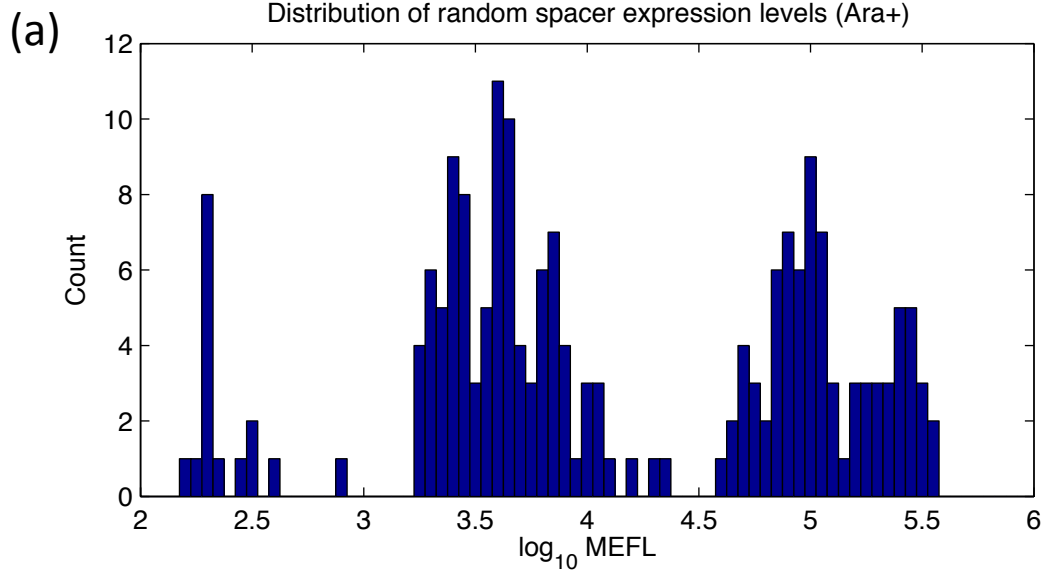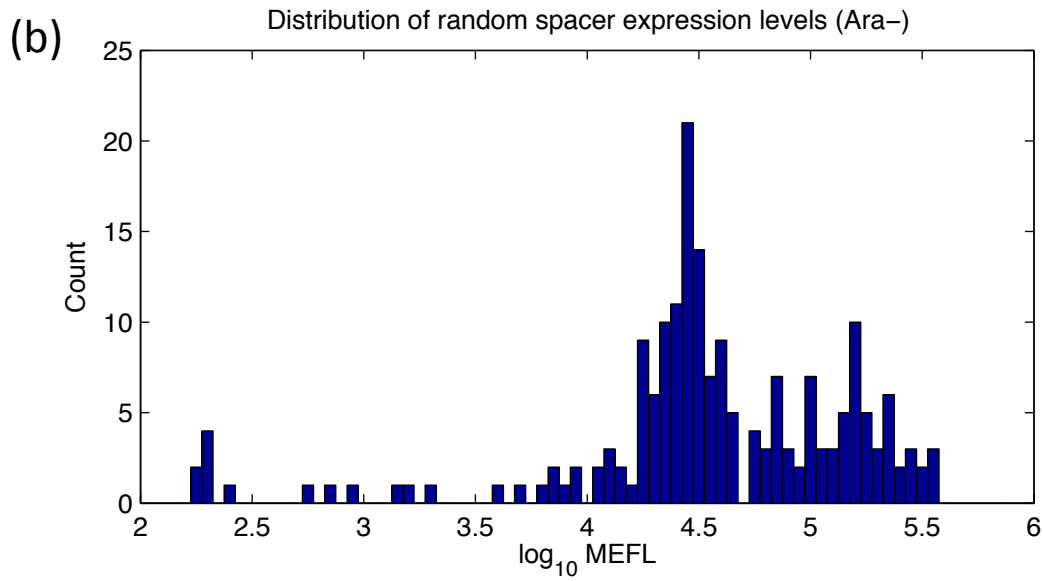

Supplement: S5 Fig — Distribution of expression levels for induced and uninduced expression levels from spacer screen for pTet, corresponding with fold-change distributions reported in Fig 1(d). (PDF) [file pone.0176013.s006.pdf]

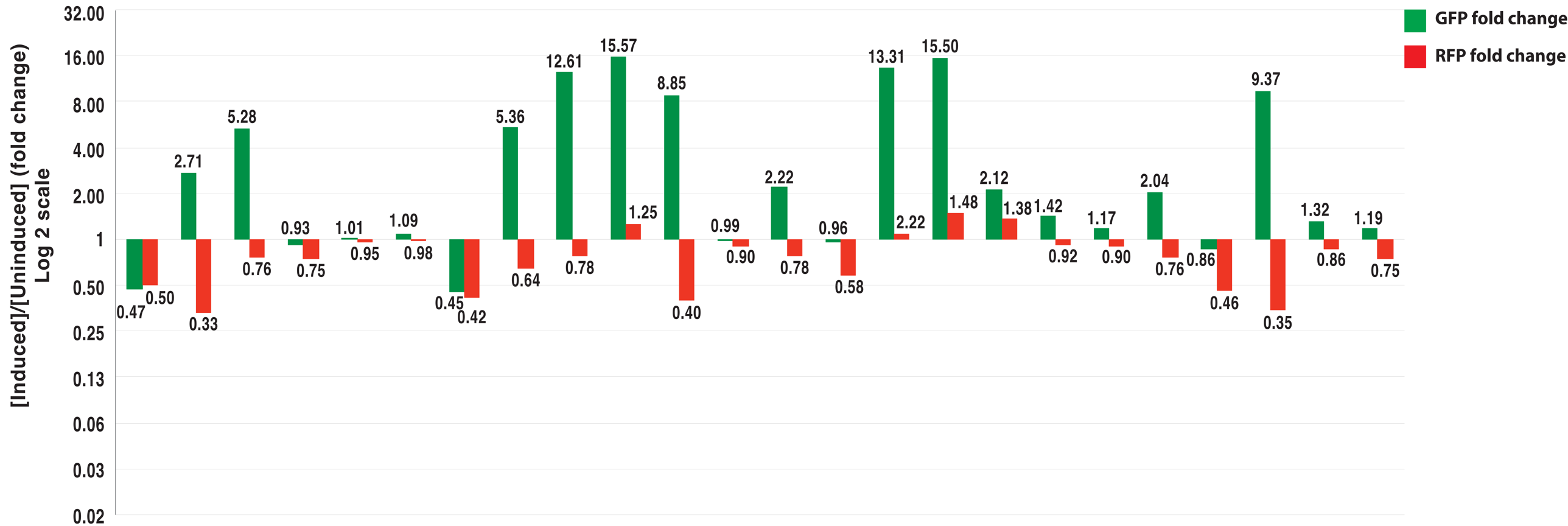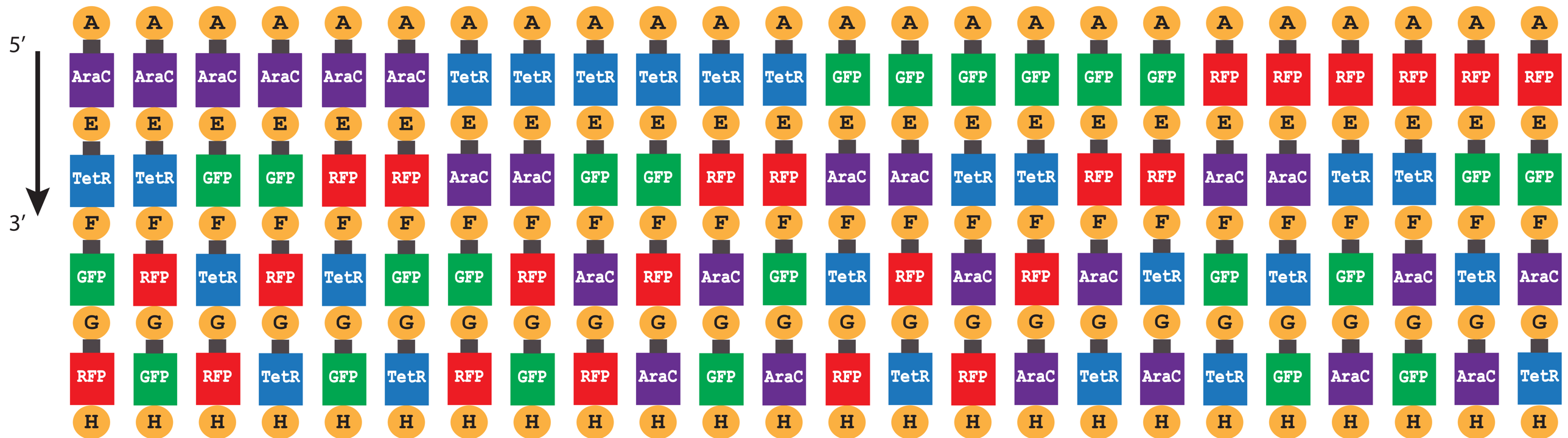

Supplement: S6 Fig — Comparison of GFP and RFP fold changes upon induction with L-arabinose of the 24-inverter permutation set in which each transcriptional unit is insulated with a different spacer from S1 Fig (INS-1 for J23100, INS-2 and INS-3 for the two pBADs, and INS-5 for pTet.). Insulation is denoted by a smaller gray box positioned between upstream MoClo fusion site and colored box denoting a transcriptional unit. Performance remains highly unreliable, indicating that it is problematic to generate spacers without consideration of their usage context. (PDF) [file pone.0176013.s007.pdf]

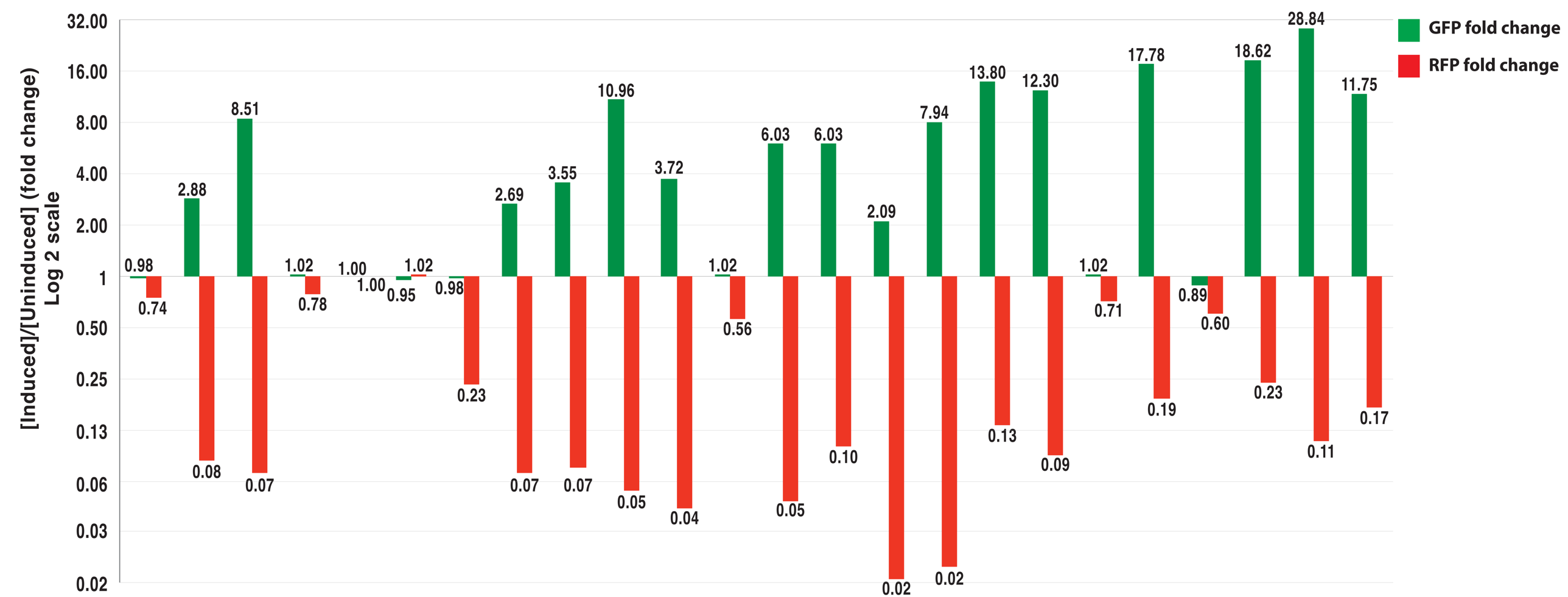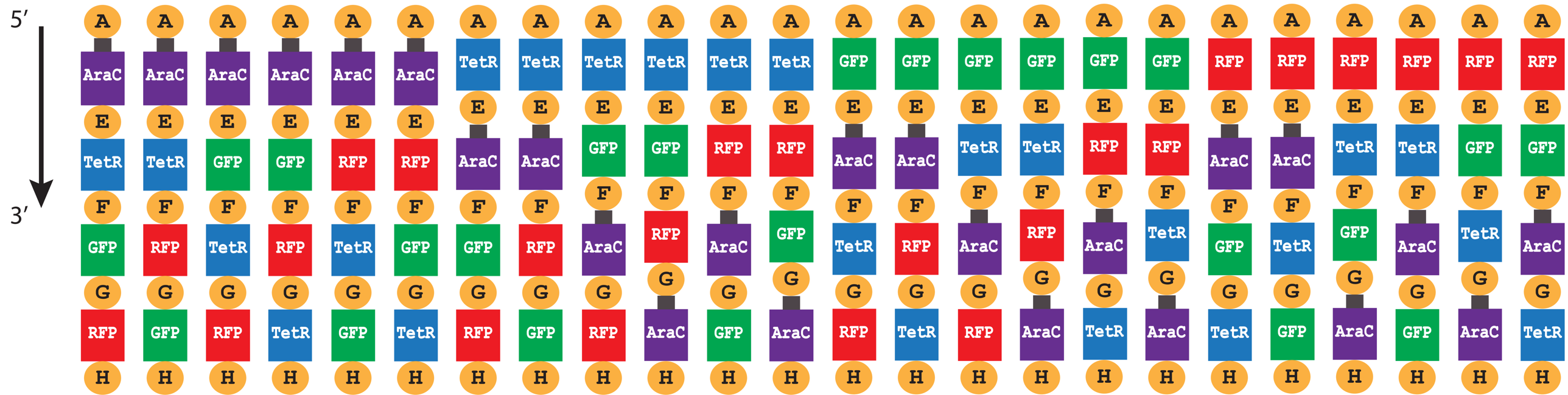

Supplement: S7 Fig — Comparison of GFP and RFP fold changes upon induction with L-arabinose of the 24-inverter permutation set in which only the J23100 promoter has been insulated with a DIS spacer. Insulation is denoted by a smaller gray box positioned between upstream MoClo fusion site and colored box denoting a transcriptional unit. Performance improves over uninsulated inverters, but is not as good as inverters with more insulators. (PDF) [file pone.0176013.s008.pdf]

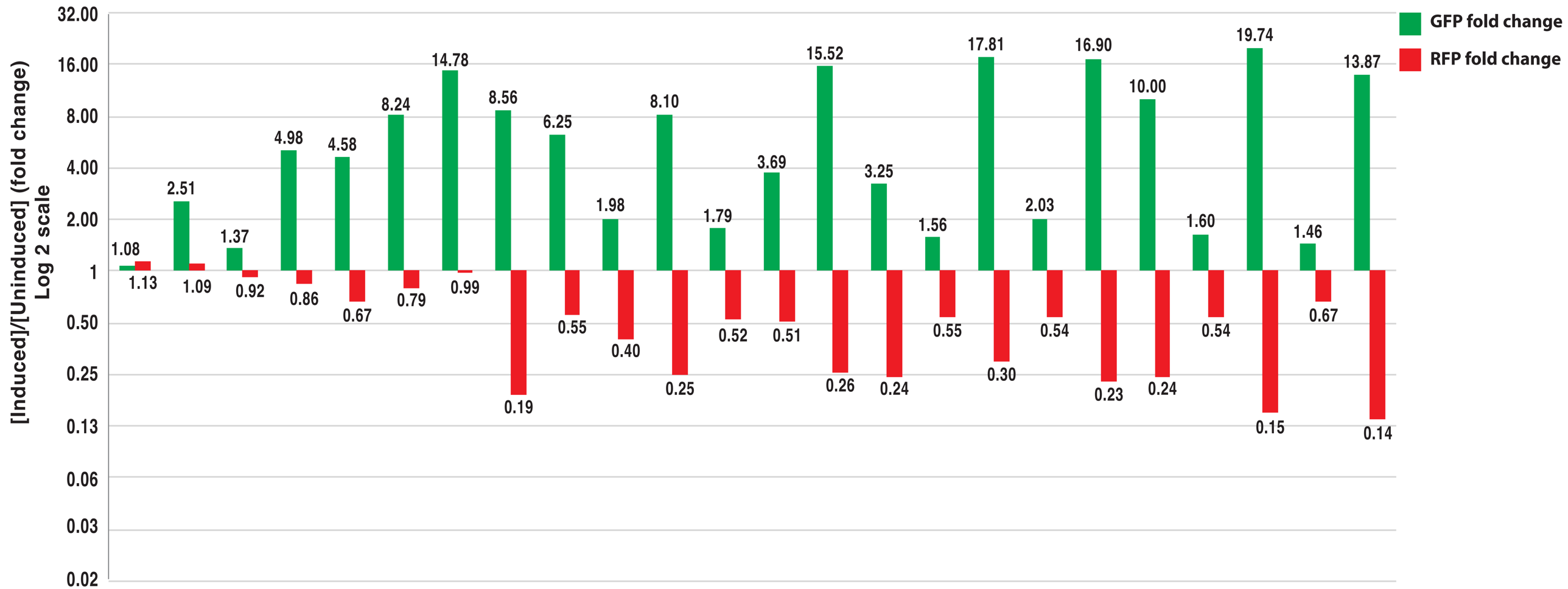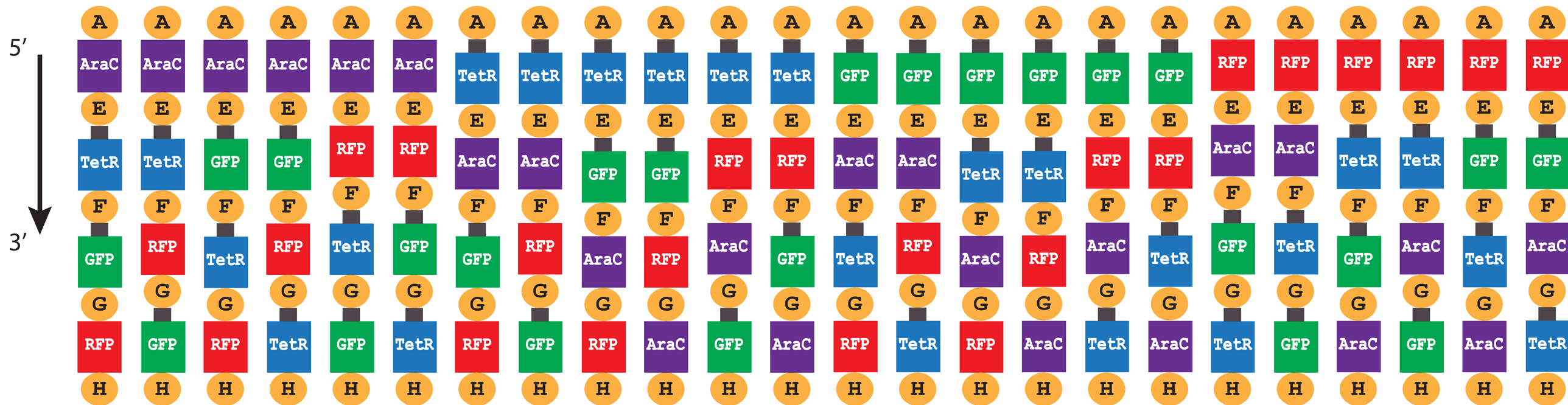

Supplement: S8 Fig — Comparison of GFP and RFP fold changes upon induction with L-arabinose of the 24-inverter permutation set in which only the two pBAD promoters have been insulated, each with a different DIS spacer. Insulation is denoted by a smaller gray box positioned between upstream MoClo fusion site and colored box denoting a transcriptional unit. Performance improves over uninsulated inverters, but is not as good as inverters with more insulators. (PDF) [file pone.0176013.s009.pdf]

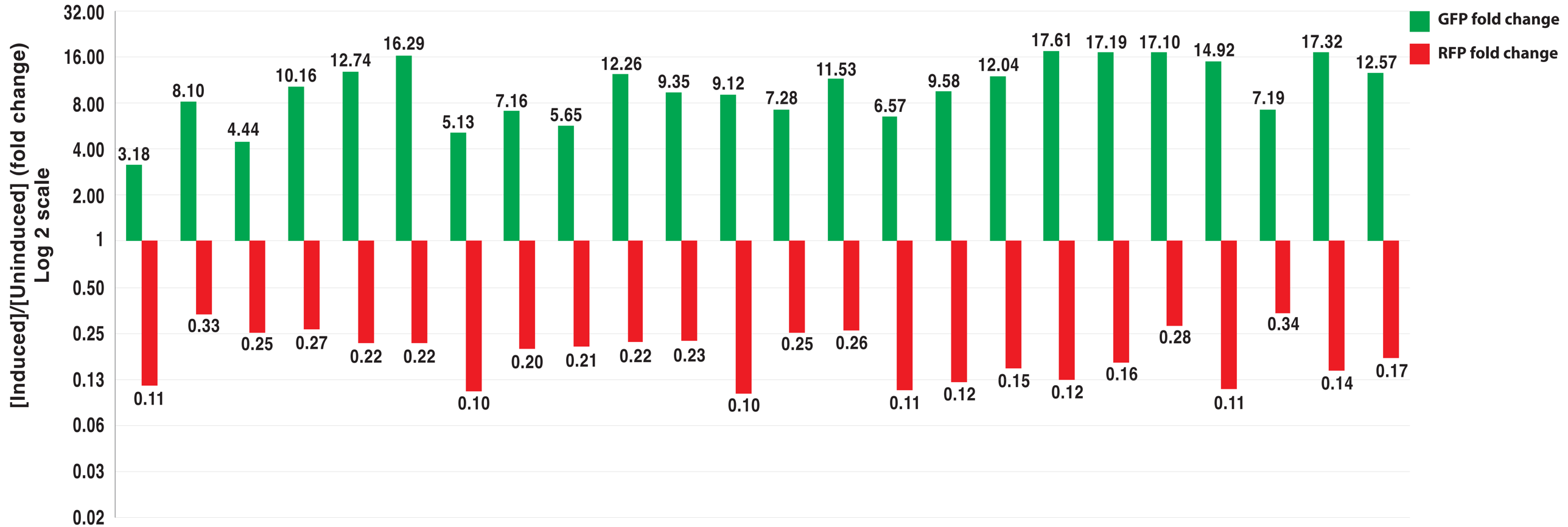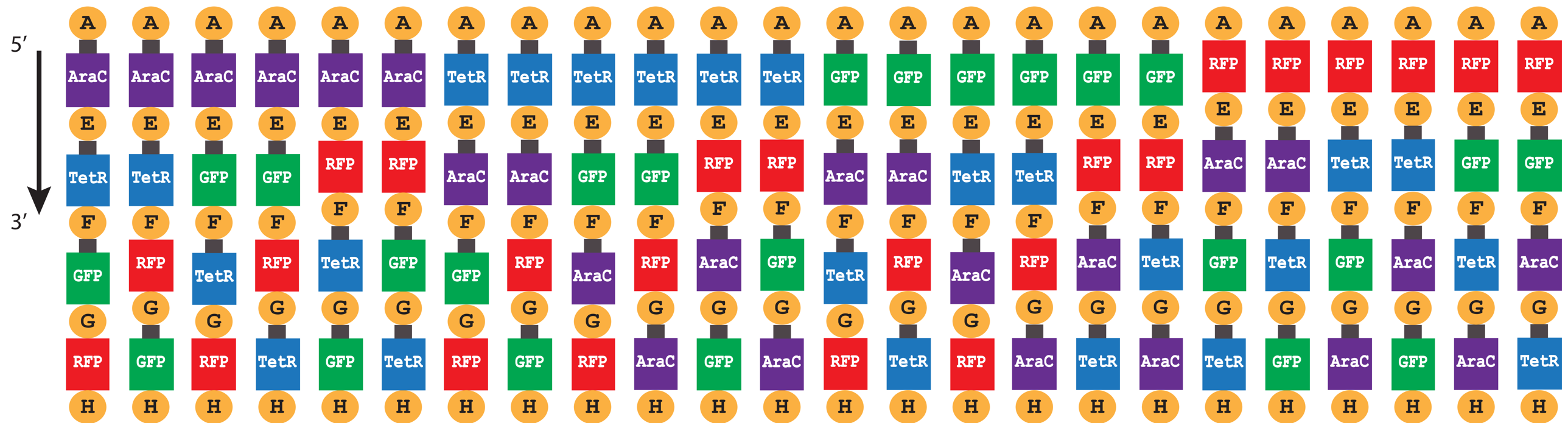

Supplement: S9 Fig — Comparison of GFP and RFP fold changes upon induction with L-arabinose of the 24-inverter permutation set in which both the J23100 and two pBAD promoters have been insulated with a DIS spacer. Insulation is denoted by a smaller gray box positioned between upstream MoClo fusion site and colored box denoting a transcriptional unit. Performance improves over a single insulator inverter, but is not as good as insulating all promoters, particularly for change in RFP expression levels. (PDF) [file pone.0176013.s010.pdf]
