# Supplementary material for: Reducing DNA context dependence in bacterial promoters
Source: PLoS One. 2017 Apr 19;12(4):e0176013. doi: 10.1371/journal.pone.0176013 (PMC5396932; doi:10.1371/journal.pone.0176013)
Supplement: S3 Fig — A preliminary test of the effect of insulator length on expression was performed with spacer sequences designed to several lengths (12 nt, 24 nt, and 36 nt) by screening random DNA base composition resembling the intergenic bacterial DNA base composition for tandem promoter elements [21] and absence of secondary structures [22, 23] that could interfere with gene expression and promoter consensus sequences. Two such spacers were inserted at the 5’ end of four different promoter-RFP expression cassettes, making eight combinations per spacer length, and fluorescent expression from each measured in triplicate. Error bars show +/- two std.dev. of mean fluorescence. While major variations in expression level between spacer pairs for a promoter were observed for 12 nt and 24 nt, only relatively low variation between pairs was observed for the 36 nt, suggesting this length may be sufficient to insulate promoters from changes in upstream sequence. (PDF) [file pone.0176013.s004.pdf]

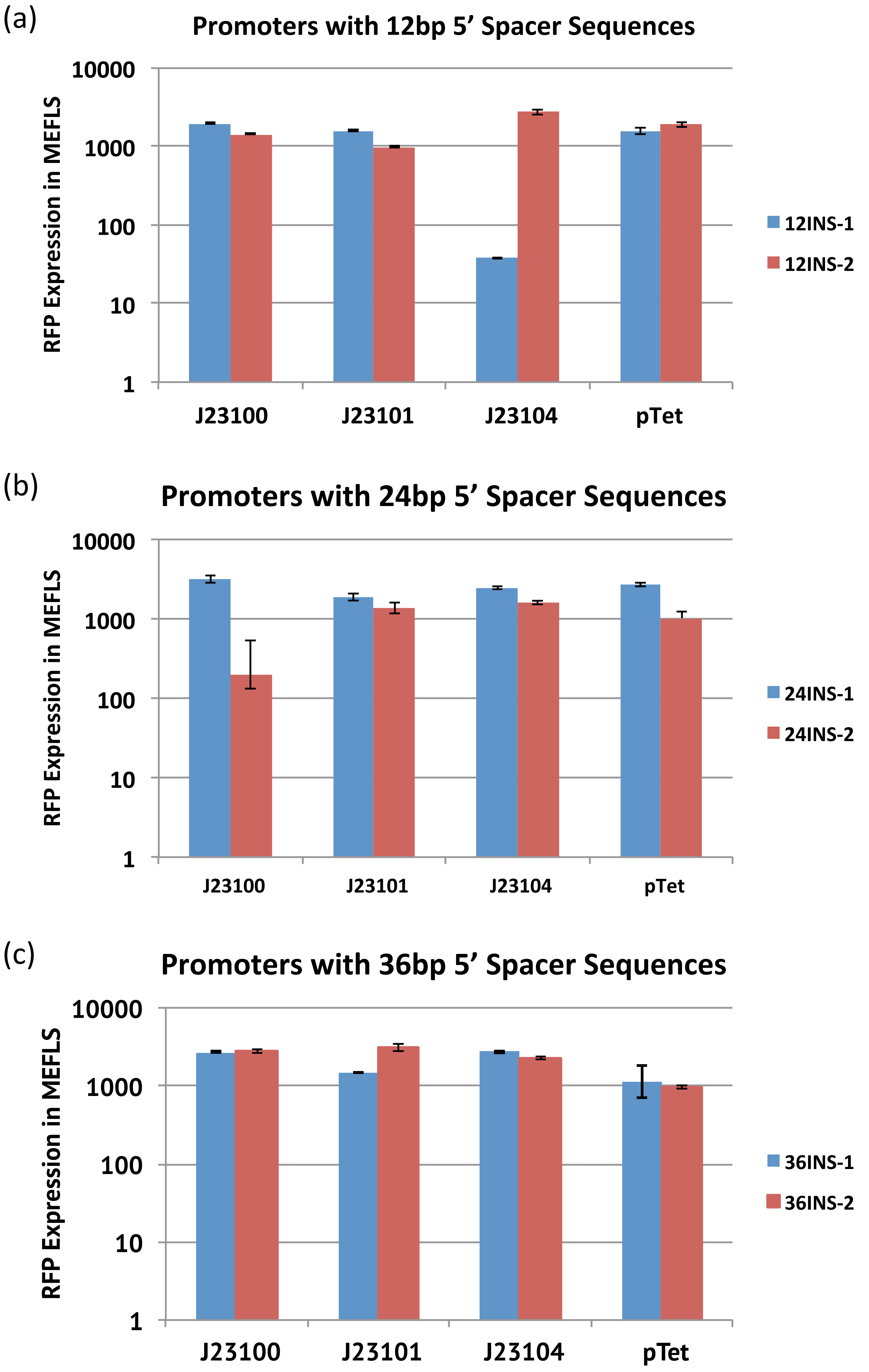

(a)
Promoters with 12bp 5' Spacer Sequences
RFP Expression in MEFLS
10000
1000
100
10
1
J23100
J23101
J23104
pTet
12INS-1
12INS-2
(b)
Promoters with 24bp 5' Spacer Sequences
RFP Expression in MEFLS
10000
1000
100
10
1
J23100
J23101
J23104
pTet
24INS-1
24INS-2
(c)
Promoters with 36bp 5' Spacer Sequences
RFP Expression in MEFLS
10000
1000
100
10
1
J23100
J23101
J23104
pTet
36INS-1
36INS-2
